# The Cancer/Testes (CT) Antigen HORMAD1 promotes Homologous Recombinational DNA Repair and Radioresistance in Lung adenocarcinoma cells

**DOI:** 10.1038/s41598-018-33601-w

**Published:** 2018-10-17

**Authors:** Yanzhe Gao, Jordan Kardos, Yang Yang, Tigist Y. Tamir, Elizabeth Mutter-Rottmayer, Bernard Weissman, Michael B. Major, William Y. Kim, Cyrus Vaziri

**Affiliations:** 1 Department of Pathology and Laboratory Medicine, University of North Carolina at Chapel Hill, 614 Brinkhous-Bullitt Building, Chapel Hill, NC 27599 USA; 2 Lineberger Comprehensive Cancer Center, Curriculum in Genetics and Molecular Biology, and Department of Biochemistry and Biophysics, University of North Carolina, Chapel Hill, NC 27599 USA; 3 Department of Cell Biology and Physiology, Lineberger Comprehensive Cancer Center, University of North Carolina at Chapel Hill, Chapel Hill, NC 27599 USA

## Abstract

The Cancer/Testes (CT) Antigen HORMAD1 is germ cell-restricted and plays developmental roles in generation and processing of meiotic DNA Double Strand Breaks (DSB). Many tumors aberrantly overexpress HORMAD1 yet the potential impact of this CT antigen on cancer biology is unclear. We tested a potential role of HORMAD1 in genome maintenance in lung adenocarcinoma cells. We show that HORMAD1 re-distributes to nuclear foci and co-localizes with the DSB marker γH2AX in response to ionizing radiation (IR) and chemotherapeutic agents. The HORMA domain and C-term disordered oligomerization motif are necessary for localization of HORMAD1 to IR-induced foci (IRIF). HORMAD1-depleted cells are sensitive to IR and camptothecin. In reporter assays, Homologous Recombination (HR)-mediated repair of targeted ISce1-induced DSBs is attenuated in HORMAD1-depleted cells. In Non-Homologous End Joining (NHEJ) reporter assays, HORMAD1-depletion does not affect repair of ISce1-induced DSB. Early DSB signaling events (including ATM phosphorylation and formation of γH2AX, 53BP1 and NBS1 foci) are intact in HORMAD1-depleted cells. However, generation of RPA-ssDNA foci and redistribution of RAD51 to DSB are compromised in HORMAD1-depleted cells, suggesting that HORMAD1 promotes DSB resection. HORMAD1-mediated HR is a neomorphic activity that is independent of its meiotic partners (including HORMAD2 and CCDC36. Bioinformatic analysis of TCGA data show that similar to known HR pathway genes HORMAD1 is overexpressed in lung adenocarcinomas. Overexpression of HR genes is associated with specific mutational profiles (including copy number variation). Taken together, we identify HORMAD1-dependent DSB repair as a new mechanism of radioresistance and a probable determinant of mutability in lung adenocarcinoma.

## Introduction

Aberrant gene expression is a hallmark of tumor cells and accounts for many phenotypes that characterize cancer. ‘Cancer/Testes (CT) Antigens’ represent an interesting group of gene products that are aberrantly expressed at high levels in many cancer cells, yet whose normal distribution is primarily germ cell-restricted^1^. The first CT antigen identified was detected in melanoma cells based on its recognition by cytolytic T lymphocytes from the same patient^2^. That CT antigen (now designated Melanoma Antigen-A1 or MAGE-A1) belongs to a larger family of *MAGE* genes whose encoded proteins are expressed in many types of cancer including lung, breast, skin, lymphoma and many others^1,3,4^. Hundreds of proteins have been designated CT antigens^5^ (http://www.cta.lncc.br/). There are at least 100 CT antigen families, many of which have multiple members. Diverse tumors express CT antigens and every individual cancer expresses a unique repertoire of the CT proteins.

CT antigens have received considerable attention as potential targets for immunotherapy^6^, but it is increasingly apparent that these proteins also possess biological activities, confer tumorigenic phenotypes and contribute directly to disease pathology^1,7^. A recent multi-dimensional screen identified numerous CT antigens that contribute directly to tumor cell viability or drive tumorigenic signaling pathways such as HIF, WNT or TGFβ^8^. The active participation of many CT antigens in cancer cell biology may explain the poor prognosis of many cancer patients whose tumors express these proteins at high levels^9–11^.

We recently identified the CT antigen MAGE-A4 as a direct binding partner and stabilizer of a DNA repair protein, RAD18 in several cancer cell lines including lung adenocarcinoma^12^. RAD18 is an apical component of the Trans-Lesion Synthesis (TLS) pathway – a specialized mode of DNA synthesis that employs damage-tolerant and error-prone DNA polymerases^13–16^. Many cancer cells rely on the MAGE-A4-RAD18 signaling axis to sustain ongoing DNA synthesis and S-phase progression following genotoxic challenge^12^. During carcinogenesis neoplastic cells must endure harsh DNA-damaging environments and tolerate DNA replication stress from metabolic sources (e.g. reactive oxygen species, or ROS) and oncogenes while simultaneously acquiring the genetic changes that fuel multi-step tumor progression. TLS allows cells to tolerate both ROS- and oncogene-induced DNA damage^17,18^. Therefore, MAGE-A4-dependent TLS activation provides a potential mechanism to explain DNA damage tolerance and mutability, two important enabling characteristics of cancer cells. Altered genome maintenance capacity/efficiency may also explain why CT antigen expression in tumors is often associated with chemoresistance^19^.

Based on our identification of a role for MAGE-A4 in genome maintenance, we hypothesized that additional CT antigens might help sustain cancer cells by promoting DNA repair. Potentially consistent with our hypothesis, several other studies have suggested effects of CT antigens on genome stability^7,20,21^. Accordingly we took a candidate gene approach to investigate possible connections between CT antigens and DNA repair. We considered two particular CT antigens HORMAD1 and HORMAD2 as DNA repair mediators in cancer cells for reasons described below.

HORMAD1 and HORMAD2 belong to a family of proteins characterized by a HORMA (Hop1, REV7, MAD2) domain that is present in several DNA repair and cell cycle factors^22,23^. mHormad1 (originally termed Newborn Ovary HORMA protein or ‘Nohma’) was first identified in an *in silico* subtractive screen for mouse genes preferentially expressed in newborn ovaries^24^. In subsequent work, HORMAD1 and the related HORMAD2 protein were shown to be preferentially associated with unsynapsed chromosome axes throughout meiotic prophase^25^. In particular, the accumulation of HORMADs on chromosomal axes correlates with sites of high checkpoint-kinase ATR activity and is inversely correlated with SC formation, suggesting that SC formation directly or indirectly promotes depletion of HORMADs from chromosome axes^25^.

The development of *Hormad1* and *Hormad2* knockout mice has revealed the important roles of the HORMADs in meiosis. *Hormad1*-deficient mice are infertile and have extensive defects in homologous pairing and synapsis^26^. Mechanistically, *mHormad1* appears to ensure availability of processed DSBs for successful homology search, normal synaptonemal-complex formation and efficient recruitment of ATR to unsynapsed chromatin^27^. Synaptonemal-complex formation in turn promotes depletion of Hormad1 from chromosome axes allowing progression through meiotic prophase checkpoints^27^.

Studies with *Hormad2*^−/−^ knockout mice have shown that Hormad2 is also required for the accumulation of ATR along unsynapsed chromosomal axes, and constitutes an asynapsis surveillance mechanism^28^.

In addition to HORMAD1/2, several other proteins involved in the meiotic chromosomal axis and the synaptonemal complex have normal developmental roles in generating and processing of meiotic DNA double strand breaks (DSB)^29,30^, and are frequently expressed at high levels in tumor cells^5,31^. However, the extent to which these CT antigens impact genome maintenance in cancer cells has received relatively little attention. In this report we investigate possible roles of CT antigens in genome maintenance in cancer cells and identify a new role for HORMAD1 in facilitating DNA repair.

## Results

### HORMAD1 is recruited to IRIF

**Figure 1:**
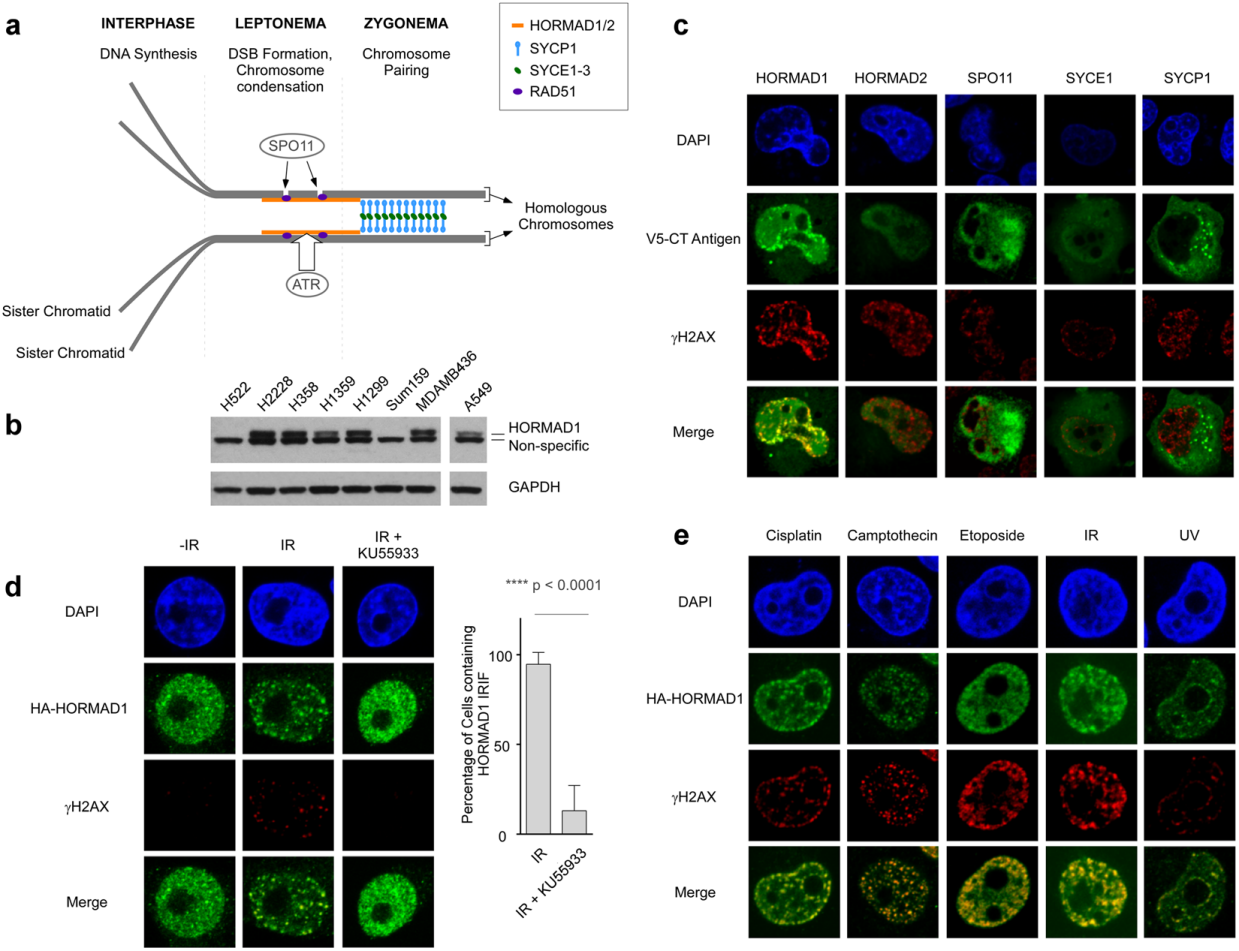
HORMAD1 redistributes to nuclear foci and co-localizes with the DNA DSB marker γH2AX in genotoxin-treated cancer cells. **(a)** Illustration depicting spatiotemporal organization of various CT antigens including HORMAD1, HORMAD2, SPO11, SYCE1 and SYCP1 during prophase I of meiosis (adapted from Bolcun-Filas and Schimenti, 2012). At the first meiotic division homologous chromosomes are joined via at least one crossover during the first prophase. Cross-overs are mediated via homologous recombination (HR) between the paired (homologous) chromosomes and the HR process is initiated via SPO11-induced DSB. A homology search juxtaposes the homologous chromosomes along their lengths and recombination is facilitated by the formation of the chromosome axis and the synaptonemal complex (SC). HORMADs associate with the unsynapsed chromosome axes and promote DNA DSB formation by the Spo11 endonuclease. **(b)** Immunoblot showing relative levels of HORMAD1 in various lung adenocarcinoma (H1299, A549, H2228, H358, H1359) and breast cancer (SUM159, MDA-MB436) cell lines. Please note that the protein sample in the A549 lane was from the same gel and immunoblot used to analyze all the other samples. An intervening ‘empty’ lane was excised from the digital image. **(c)** Plasmids encoding V5 epitope-tagged HORMAD1, HORMAD2, SPO11, SYCE1 and SYCP1 were transfected into H1299 lung carcinoma cells. 48 h later the transfected cells were irradiated (10 Gy) or sham-treated and 1 h later the subcellular distribution of each CT antigen in relation to γH2AX was analyzed by confocal microscopy. We used the microscopy image analysis software IMARIS (see Materials and Methods) to empirically measure and confirm that HA-HORMAD1 co-localized with γH2AX as shown in Supplementary Fig. S1. **(d)** HA-HORMAD1 was expressed in H1299 cells using a recombinant adenovirus. 24 h post-infection, some cultures were treated with 20 μM KU55933 for 1 h. Control and KU55933-treated cells were conditionally irradiated (10 Gy) and 1 h later the subcellular distribution of HA-HORMAD1 in relation to γH2AX was analyzed by confocal microscopy. The bar graph summarizes results of two independent experiments in which 100 cells were scored for HORMAD1 IRIF in the absence and presence of ATM inhibitor. Error bars indicate the standard deviation of results from two independent experiments. Quantification of Hormad1-53BP1 colocalization is presented in Supplementary Fig. 1B. (**e**) HA-HORMAD1 was expressed in H1299 cells using a recombinant adenovirus. 24 h post-infection, cultures were treated with various DNA-damaging agents. The subcellular distribution of HA-HORMAD1 in relation to γH2AX was analyzed by confocal microscopy at specific times (indicated in parentheses) after each genotoxin treatment: 5 μg/ml Cisplatin (6 h), 100 nM Camptothecin (6 h), 100 μM Etoposide (6 h), 10 Gy IR (1 h), 20 J/m^2^ UVC (6 h). For each genotoxin treatment the treatment conditions selected (dose, time) are ones that we and others have shown are associated with DSB formation. The average Pearson’s correlation coefficients for co-localization of Hormad1 with γH2AX in response to different treatments are as follows: 0.7 for cisplatin, 0.58 for Camptothecin, 0.72 for Etoposide, 0.67 for IR, and 0.53 for UV. The images shown in panel E are representative of nuclei with focal patterns that were observed in 2 independent experiments.

The CT Antigens HORMAD1, HORMAD2, SPO11, SYCE1 and SYCP1 participate in meiotic DSB induction and processing in germ cells^29^. Figure 1a indicates the spatial-temporal distribution of these CT antigens during prophase I of the first meiotic division. Because HORMAD1, HORMAD2, SPO11, SYCE1 and SYCP1 are frequently mis-expressed in tumors^31^ we sought to investigate the impact of these CT antigens on DSB processing in cancer cells. We selected H1299 lung adenocarcinoma cells for these initial experiments for several reasons:

(1) HORMAD1 is frequently overexpressed in lung cancer (as described later in this report). (2) HORMAD1 protein levels in H1299 cells were equivalent to those in other HORMAD1-positive cancer cells (Fig. 1b) including lung adenocarcinoma and Triple Negative Breast Cancer (TNBC) cell lines (a setting in which effects of HORMAD1 on DNA repair have previously been reported)^32^. (3) The DNA replication, DNA repair and cell cycle parameters of H1299 cells have been extensively characterized by us and many other researchers in the genome maintenance field^33–36^. (4) We recently showed that H1299 cells deploy at least one CT antigen (MAGE-A4) to promote genome maintenance^12^.

Our immunoblot analysis of HORMAD1 expression in a panel of cancer cells (Fig. 1b) identified several HORMAD1-positive and HORMAD1-deficient cell lines. This information guided our selection of cells for subsequent experiments in which we studied endogenous HORMAD1 function (by depleting the protein from HORMAD1+ cells), or analyzed consequences of ectopic expression (performed both in HORMAD1+ and HORMAD1− cells).

As an initial test of whether CTAs participate in genome maintenance we asked whether HORMAD1, HORMAD2, SPO11, SYCE1 and SYCP1 redistributed to nuclear foci in response to IR-induced DNA double strand breaks. Interestingly, HORMAD1 (but not any of the other CTA tested) was redistributed to IRIF and co-localized with γH2AX in irradiated H1299 lung carcinoma cells (Fig. 1c). The extent to which HORMAD1 co-localized with γH2AX was empirically quantified using the microscopy image analysis software IMARIS to calculate the Pearson’s correlation coefficient using the method of Dunn *et al*.^37^ as described in Supplementary Fig. S1a.

ATM-family kinases mediate DSB-induced γH2AX phosphorylation, facilitate the assembly of various genome maintenance proteins at IRIF in somatic cells, and also regulate HORMAD1 in meiosis^29,38^. Therefore, we used a pharmacological inhibitor (KU55933) to test the potential contribution of ATM signaling to HORMAD1 regulation in cancer cells. As shown in Fig. 1d, pretreatment of H1299 cells with KU55933 abrogated both IR-induced γH2AX foci and HORMAD1 foci. We also examined the effect of ATM inhibition on DSB-induced localization of HORMAD1 in relation to 53BP1, a DNA repair protein which forms IRIF in an ATM-independent manner^39^. As shown in Supplementary Fig. 1b, IR-induced 53BP1 foci were refractory to ATM inhibition although co-localization of 53BP1 with HORMAD1 was abrogated by ATM inhibition. We conclude that ATM signaling promotes HORMAD1 IRIF.

In IR-treated cells DSBs are generated directly and in a cell cycle-independent fashion. However, DSB can also arise secondary to collisions between DNA replication forks and DNA lesions from diverse genotoxic agents including chemotherapeutic agents and environmental exposures. Therefore we asked whether HORMAD1 redistributes to sites of DNA damage in response to other genotoxic agents. As shown in Fig. 1e, HORMAD1 co-localized with the DSB marker γH2AX in response to cisplatin (a chemotherapeutic drug that causes inter- and intra-strand DNA crosslinks), camptothecin and etoposide (which inhibit Topoisomerases I and II respectively) and ultraviolet (UV) radiation (which causes cyclobutane pyrimidine dimers and photoproducts). Therefore, HORMAD1 is recruited to DSB from diverse sources. HORMAD1 IRIF were also readily detectable in other cancer cell lines including A549 lung adenocarcinoma, and U2OS osteosarcoma (Supplementary Fig. S1c). We conclude that HORMAD1 is actively recruited to the vicinity of DSB in an ATM-dependent manner.

### Structure/function analysis of HORMAD1

**Figure 2:**
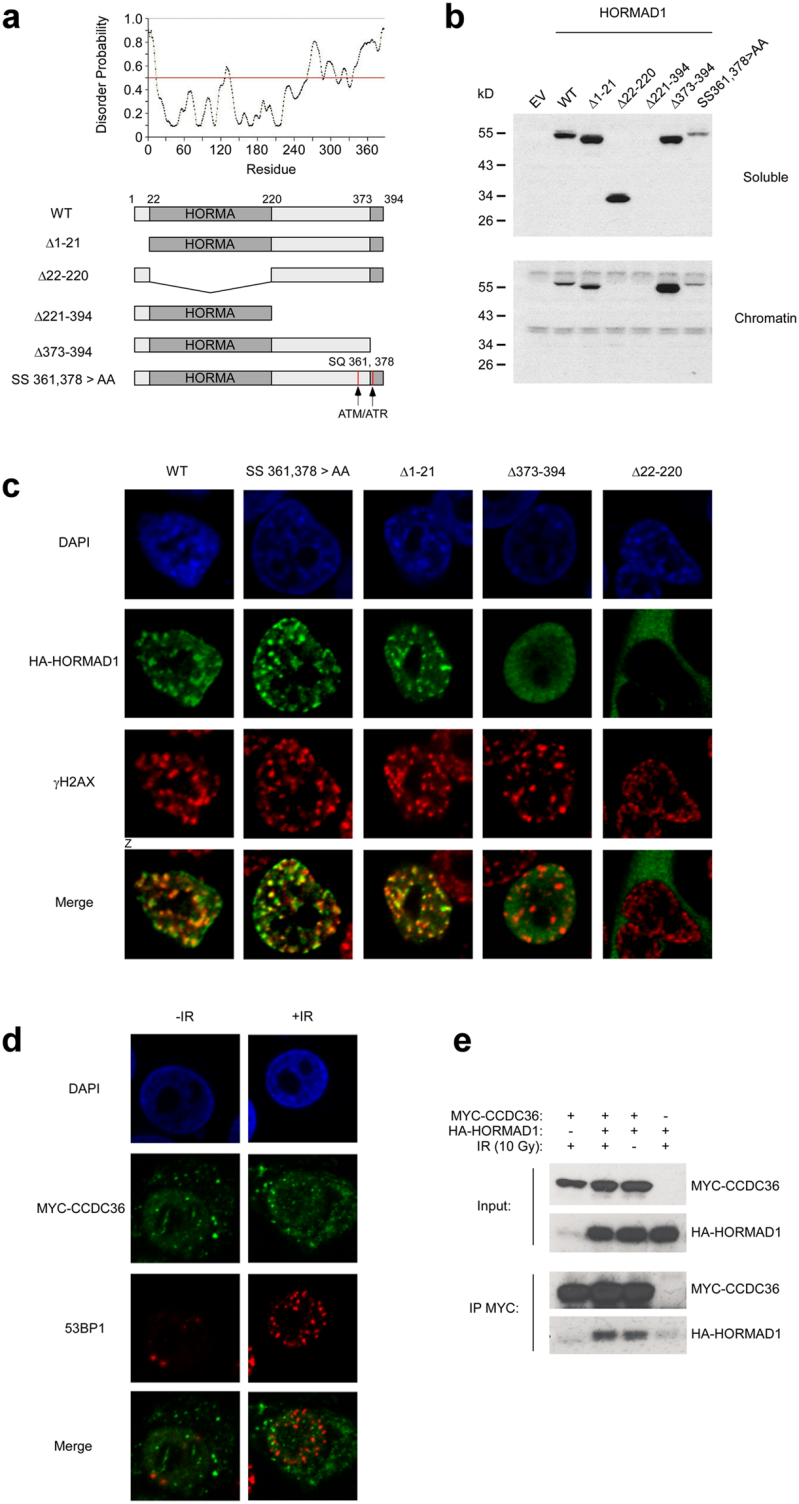
Defining functional domains of HORMAD1 in the DNA damage response. **(a)** Disorder probablility profile of HORMAD1 (predicted using the Protein Disorder Prediction System at http://prdos.hgc.jp/cgi-bin/top.cgi) and summary of deletion mutants used in this study. **(b)** H1299 cells were infected with adenovirus vectors encoding HA epitope-tagged wild-type (WT) and mutant forms of HORMAD1. 24 h later, cells were biochemically fractionated and the resulting soluble and chromatin extracts were analyzed by SDS-PAGE and immunoblotting with anti-HA antibody. The immunoblot shows relative expression of WT and mutant forms of HORMAD1 in soluble and chromatin compartments. **(c)** H1299 cells were infected with adenovirus vectors encoding HA epitope-tagged wild-type (WT) and mutant forms of HORMAD1. 24 h later, cells were analyzed for HORMAD1 distribution using confocal microscopy. The average Pearson’s correlation coefficients for co-localization of HORMAD1 with γH2AX were 0.50 for HORMAD1 WT, 0.57 for HORMAD1 SS 361,378 > AA, 0.53 for HORMAD1 Δ1-21, −0.23 for HORMAD1 Δ22-220, and 0.007 for Δ373-394. These results indicate that the HORMA domain and the extreme C-terminal amino acids of HORMAD1 are necessary for co-localization of HORMAD1 with γH2AX-containing IRIF. **(d)** H1299 cells were transfected with a plasmid encoding MYC-CCDC36. 24 h post-transfection, some cultures were irradiated (10 Gy) and 1 h later the subcellular distribution of CCDC36 in relation to 53BP1 was was analyzed by confocal microscopy. **(e)** H1299 cells were transfected with a plasmid encoding MYC-CCDC36 individually or in combination with an HA-HORMAD1 expression vector. 48 h later, cells were treated with IR (10 Gy) or were sham-irradiated. 1 h after irradiation cells were harvested and extracts were immunoprecipitated using MYC antibodies. MYC immune complexes or total ‘Input’ fractions were analyzed by SDS-PAGE and immunoblotting with anti-HA and anti-MYC antibodies.

The spatiotemporal regulation of mHormad1 in germ cells, and the roles of Hormad1 and its binding partners in establishing the meiotic chromosome axis have been studied extensively in mice. However, nothing is known regarding the regulation of HORMAD1 (or its putative functions) in cancer cells (in any species). We performed structure-function analyses to define mechanisms of HORMAD1 regulation by DSB signaling in cancer cells. We used existing knowledge of mHormad1 domains and their meiotic functions to perform mutational analysis of HORMAD1. Our mutational analyses were also guided by the disorder probability profile of HORMAD1 (Fig. 2a) since disordered regions typically contain motifs critical for protein function. We generated HORMAD1 mutants lacking the N-terminus (HORMAD1 Δ1-21), the HORMA domain (HORMAD1 Δ22-220), the disordered C-terminal portion of the protein immediately flanking the HORMA domain (Δ221-394), the extreme C-terminal closure motif peptide (HORMAD1 Δ373-394), and two consensus ATM/ATR sites (HORMAD1 SS 361,378 > AA) (Fig. 2a).

With the exception of HORMAD1 Δ221-394, all mutants were readily expressed at levels comparable to WT HORMAD1 in H1299 cells (Fig. 2b). Similar to WT HORMAD1, the N-terminal HORMAD1 Δ1-21 mutant bound chromatin (Fig. 2b) and formed γH2AX-colocalizing IRIF (Fig. 2c). The HORMA domain deletion mutant HORMAD1 Δ22-220 did not bind chromatin and was excluded from the nucleus (Fig. 2b,c). Interestingly, the HORMAD1 mutant lacking the C-terminal closure peptide (HORMAD1 Δ374-394) partitioned to the nucleus and bound chromatin, yet did not form IRIF. ATM site mutations did not affect subcellular distribution, chromatin binding, or IRIF formation (Fig. 2b,c). Taken together, our mutational analyses show that the effects of ATMi on HORMAD1 IRIF cannot be attributed to direct phosphorylation of HORMAD1 at 361 and 378. Moreover, HORMAD1 domains that are necessary for meiotic functions (HORMA motif, C-terminal closure peptide) are also necessary for responsiveness to DSB in cancer cells.

Some meiotic functions of HORMAD1 are dependent on a related CT antigen, its oligomerization partner HORMAD2. However, HORMAD2 is not expressed in H1299 cells (Supplementary Fig. S2) and therefore the IRIF activity of HORMAD1 in cancer cells is HORMAD2-independent. CCDC36 is another meiotic HORMAD1-binding partner^40^ and is expressed in many cancer cells including H1299 (Supplementary Fig. S2). We asked whether CCDC36 co-localizes with HORMAD1 in IRIF. As expected, ectopically co-expressed CCDC36 was co-immunoprecipitated with HORMAD1 (Fig. 2e), indicating that CCDC36 and HORMAD1 can interact in cancer cells. However, CCDC36 did not redistribute to IRIF. Therefore, HORMAD1 regulation in response to DSB is independent of its two known meiotic partners HORMAD2 and CCDC36 (and thus represents a ‘neomorphic’ activity).

### HORMAD1 promotes DSB repair and Radio-resistance

**Figure 3:**
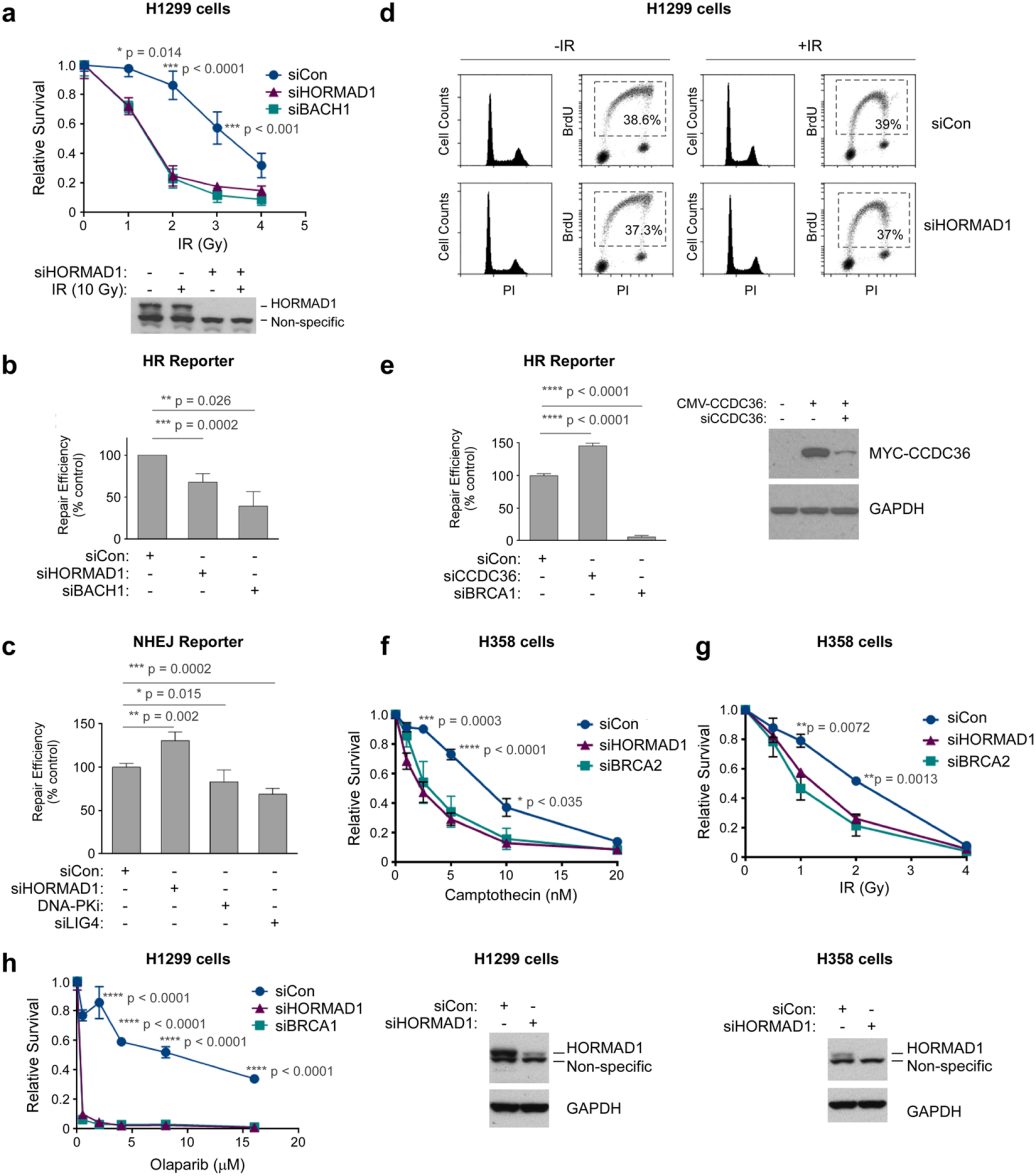
HORMAD1 promotes Homologous Recombination and DNA damage tolerance in cancer cell lines. **(a)** Replicate cultures of H1299 cells were transfected with siRNAs targeting HORMAD1 or BRCH1 (or with non-targeting control oligonucleotides). Transfected cells were treated with the indicated dose range of IR and DNA damage sensitivities were measured by clonogenic survival assays. The lower panel is an immunoblot showing relative levels of HORMAD1 expression 48 h post transfection in the siCon- and siHORMAD1-treated cells. **(b)** Replicate plates of H1299 cells harboring the stably-integrated DR-GFP reporter construct were transfected with siRNAs (against HORMAD1, BACH1, or non-targeting siRNA). 24 h later the siRNA-treated cells were transfected with an ISceI expression plasmid (to induce DSB in the DR-GFP locus) or with an empty control vector. After 24 h cells were trypsinized and GFP-expressing populations (resulting from HR-mediated reconstitution of a silent GFP allele) were enumerated by flow cytometry. Supplementary Fig. S3A shows the original flow cytometry profiles corresponding to these HR assays. In QPCR analyses of mRNA from siBACH1-transfected cells, endogenous BACH1 transcript levels were reduced by 62% relative to control (siCon) cultures. **(c)** Replicate plates of H1299 cells harboring the stably-integrated EJ5-NHEJ reporter construct were transfected with siRNAs (against HORMAD1, LIG4, or non-targeting siRNA). Some cultures were also treated with the DNA-PK inhibitor NU7441 (20 μM). 24 h later the siRNA or DNA-PKi-treated cells were transfected with an ISceI expression plasmid (to induce DSB in the stably integrated reporter locus) or with an empty control vector. After 24 h cells were trypsinized and GFP-expressing cells (arising from NHEJ-mediated reconstitution of a silent GFP allele) were enumerated by flow cytometry. In QPCR analyses of mRNA from siLIG4-transfected cells, LIG4 transcript levels were reduced by 95% relative to control (siCon) cultures. **(d)** H1299 cells were transfected with siRNAs targeting HORMAD1 or with non-targeting control oligonucleotides. 48 h later cells were treated with 10 μM BrdU for 1 h to label actively-replicating DNA. Cells were then collected and stained sequentially using a FITC-labeled anti-BrdU antibody and PI. The labeled nuclei were analyzed by flow cytometry. The BrdU-positive (S-phase) populations are indicated by the dashed quadrant. **(e)** Replicate plates of H1299 cells harboring the stably-integrated DR-GFP reporter construct were transfected with siRNAs against CCDC36, BRCA1, or with non-targeting siRNA (siCon). 24 h later the siRNA-treated cells were transfected with an ISceI expression plasmid and HR activity was measured based on enumeration of GFP-positive cells as described for panel (b) above. In QPCR analyses of mRNA from siBRCA1-transfected cells, BRCA1 transcript levels were reduced by 96% relative to control (siCon) cultures. **(f,g)** Replicate cultures of H358 cells were transfected with siRNAs targeting HORMAD1 or BRCA2 (or with non-targeting control oligonucleotides). In QPCR analyses of mRNA from siBRCA2-transfected cells, levels of the endogenous BRCA2 transcript were reduced by 92% relative to control (siCon) cultures. Transfected cells were treated with the indicated dose ranges of camptothecin (**f**) or IR (**g**) and DNA damage sensitivities were measured by clonogenic survival assays. **(h)** Replicate cultures of H1299 cells were transfected with siRNAs targeting HORMAD1 or BRCA1 (or with non-targeting control oligonucleotides). Transfected cells were treated with the indicated dose ranges of olaparib and viability was determined by clonogenic survival assays. Error bars throughout this figure represent the standard error of the mean from three independent experiments that were performed in triplicate.

The CT antigen MAGE-A4, confers tolerance of bulky DNA adducts in cancer cells^12,41^. The redistribution of HORMAD1 to IRIF suggested an analogous role for HORMAD1 in tolerance of DSB. We performed colony survival assays in control and HORMAD1-depleted H1299 cells that were exposed to a 0–10 Gy dose range of IR. As a positive control for a known HR gene we also depleted BACH1 in replicate cultures. Figure 3a shows that HORMAD1-depleted H1299 cells were IR-sensitive when compared with HORMAD1-replete cells transfected with non-targeting (siCon) siRNA. Remarkably, HORMAD1-depleted cells fully phenocopied the IR-sensitivity of BACH1-depleted cells^42^ (Fig. 3a), consistent with a role for HORMAD1 in DSB repair.

The DNA damage sensitivities revealed by colony survival assays often result from attenuation of Homologous Recombination (HR) or Non-Homologous End Joining (NHEJ) – two major pathways of DSB repair. We used reporter assays to quantify HR and NHEJ-mediated repair of ISce1-induced DSB in control (siCon) and HORMAD1-depleted H1299 cells. As expected, depleting BACH1 led to attenuation of HR activity relative to control cultures. Interestingly, HR reporter activity was also reduced (by approximately 40%) in HORMAD1-depleted cells relative to control HORMAD1-replete cultures (Fig. 3b). Similar results were obtained using two independent HORMAD1 siRNAs (Supplementary Fig. 3b). Therefore the effects of HORMAD1 siRNA on HR reporter activity are unlikely to be off-target. HORMAD1-depletion in another lung adenocarcinoma cell line (A549) also led to attenuation of HR activity as measured by DR-GFP reporter assay (Supplementary Fig. S3c).

We validated the NHEJ assay by demonstrating that depletion of LIG4 or pharmacological inhibition of DNA-PK attenuated NHEJ reporter activity relative to control cultures (Fig. 3c). In HORMAD1-depleted cells NHEJ activity was modestly but significantly increased when compared with HORMAD1-expressing cultures (Fig. 3c). Therefore, HORMAD1 promotes HR but not NHEJ. The slight increases in NHEJ activity in HORMAD1-depleted cells may result from channeling of DSB into the NHEJ pathway when HR is unavailable. Because HR is coupled to DNA replication, we considered the possibility that the reduced HR activity of HORMAD1-depleted cells was secondary to an S-phase defect. However, HORMAD1-depletion did not affect cell cycle distribution or DNA replication rates of H1299 cells in unirradiated or IR-treated cells (Fig. 3d). We conclude that the reduced HR activity of HORMAD1-depleted cells results from a specific decrease in HR and is not a secondary consequence of changes in DNA replication.

HORMAD1-mediated HR was independent of its meiotic binding partner HORMAD2 (which is not expressed in H1299 cells – see Supplementary Fig. S2). siRNA-mediated knockdown of CCDC36 (another meiotic HORMAD1-binding partner), did not attenuate HR activity (Fig. 3e). The HORMAD2- and CCDC36-independence of DSB repair in our HR assays is consistent with the hypothesis that HORMAD1 acquires neomorphic DNA repair activities when mis-expressed in cancer cells.

In colony survival assays, HORMAD1-depletion also sensitized another lung adenocarcinoma cancer cell line (H358) to DNA damage from IR (Fig. 3g) or camptothecin (Fig. 3f) – a therapeutic agent which also induces DSB. We conclude that neoplastic cells deploy HORMAD1 to activate HR and tolerate DSB.

HR-defective cells are often sensitive to PARP inhibitors^43^. As shown in Fig. 3h, HORMAD1-depleted cells were highly sensitive to the PARP inhibitor olaparib and fully recapitulated the synthetic lethality resulting from combined PARP-inhibition and BRCA1-depletion. The PARP-sensitivity of HORMAD1-depleted cells is further consistent with a role for HORMAD1 in HR.

### HORMAD1 promotes assembly of HR factors at IRIF

**Figure 4:**
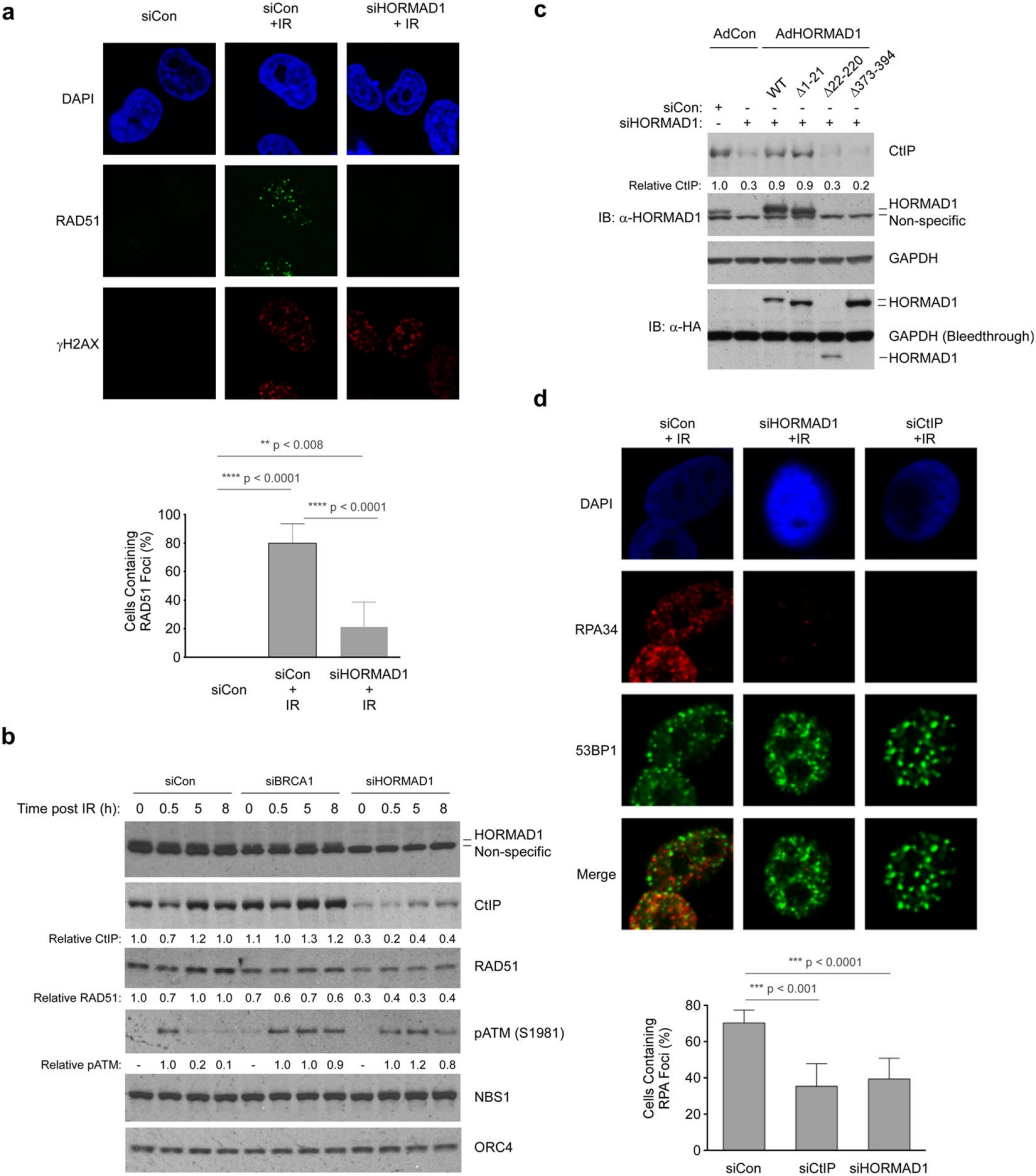
HORMAD1 promotes DSB resection to promote HR. **(a)** H1299 cells were transfected with siCon or siHORMAD1. 48 h post-transfection cells were irradiated (10 Gy) and 1 h later nuclei were analyzed for RAD51 and γH2AX distribution using confocal microscopy. The number of cells containing RAD51 IRIF was reduced to 33% in HORMAD1-depleted cells when compared with control cultures (p < 0.001). Error bars represent the standard deviation and were derived from three independent experiments. **(b)** Replicate cultures of control, HORMAD1-depleted or BRCA1-depleted cells were irradiated (10 Gy). At different times post-IR, chromatin fractions were analyzed by SDS-PAGE and immunoblotting with the indicated antibodies. **(c)** H1299 cells were reverse transfected with siCon or siHORMAD1-5′UTR (which targets the untranslated region of the endogenous HORMAD1 mRNA but not the ectopically expressed HORMAD1 transcript). 16 h post-transfection cells were infected with advenovirus vectors encoding WT and mutant forms of HORMAD1. 48 hours post infection, cells were irradiated (10 Gy) and harvested 1 h later for SDS-PAGE and immunoblotting analysis with the indicated antibodies. **(d)** H1299 cells were transfected with siCon, siHORMAD1, or siCtIP. 48 h post-transfection cells were irradiated (10 Gy) and 1 h later nuclei were analyzed for RPA34 and 53BP foci using immunofluorescence confocal microscopy. 53BP1 focus formation was not significantly affected by HORMAD1 depletion. The experiment shown here was performed in parallel with the experiment presented in Supplementary Fig. 4. Immunoblots shown in Supplementary Fig. S4 validates efficient ablation of HORMAD1 and CtIP expression by these siRNAs. The histogram shows quantification of RPA IRIF data from multiple experiments. The percentage of cells containing RPA foci is counted. Depletion of CtIP or HORMAD1 significantly reduced the RPA- positive population of IR-treated cells by approximately 50% (relative to control siRNA transfected cultures). Error bars represent the standard deviation and were derived from three independent experiments.

HR requires the coordinated and sequential recruitment of multiple DNA repair proteins at DSB. We used formation of IRIF and chromatin-binding of DNA repair proteins as assays to determine the dependencies of different HR factors on HORMAD1. As shown by confocal microscopy analyses, γH2AX-colocalized RAD51 IRIF were readily detectable in irradiated HORMAD1-replete cells (Fig. 4a). However, the number of cells containing RAD51 IRIF was reduced to 33% in HORMAD1-depleted cells when compared with control cultures (p < 0.001). Therefore, HORMAD1 facilitates HR prior to the RAD51-mediated strand invasion step.

To further pinpoint the HORMAD1-dependent phase of HR we performed immunoblotting to examine the effects of HORMAD1-depletion on the chromatin-binding of various genome maintenance proteins.

As a positive control we also depleted BRCA1, an HR factor which stimulates RAD51 activity via direct interactions. Consistent with our confocal microscopy analyses (Fig. 4a), levels of chromatin-bound RAD51 were attenuated in BRCA1- and HORMAD1-depleted cells (Fig. 4b). Interestingly, the IR-inducible phosphorylation of ATM at S1981 was more persistent in both BRCA1- and HORMAD1-depleted cells (Fig. 4b), indicative of reduced DSB repair when compared with control cells. The defective chromatin-binding of RAD51 and persistence of ATM phosphorylation in both BRCA1- and HORMAD1-depleted cells are fully consistent with a role for HORMAD1 in promoting HR.

CtIP plays a key role in resection of DSB into ssDNA species that invade the sister chromatid template in a BRCA1/RAD51-dependent manner. CtIP-mediated DSB resection occurs upstream of BRCA1 in the HR pathway and as expected, CtIP levels on chromatin were unaffected by BRCA1 depletion. Interestingly however we noticed that the basal and IR-inducible chromatin-binding of CtIP was reduced in HORMAD1-depleted cells (Fig. 4b). The reduced levels of chromatin-bound CtIP indicated a role for HORMAD1 in DSB resection.

An ectopically expressed siRNA-resistant form of full-length HORMAD1 (harboring silent mutations) corrected the CtIP defect of HORMAD1-depleted cells (Fig. 4c). Therefore, the effect of HORMAD1-depletion on CtIP regulation cannot be attributed to off-target effects of HORMAD siRNA. We also generated siRNA-resistant versions of the HORMAD1 mutants described in Fig. 2a and tested these for CtIP regulation in cells depleted of endogenous HORMAD1. As shown in Fig. 4c, the HORMAD1Δ1-21 mutant (lacking 21 amino-terminal amino acids) supported chromatin-binding of CtIP to the same degree as full-length HORMAD1. Interestingly however, the HORMAD1 Δ221-394 and HORMAD1 Δ372-394 deletion mutants (which lack 173 and 21 C-terminal amino acids respectively) were unable to sustain chromatin-binding of CtIP (Fig. 4c). Therefore, based on our mutational analyses, the structural requirements for HORMAD1 redistribution to IRIF and HORMAD1-dependent regulation of CtIP are identical. Notably, these structure-function analyses show that a 21 amino acid C-terminal region of HORMAD1 which is required for interaction with meiotic partners in germ cells^44^ is also required to redistribute HORMAD1 to IRIF and to sustain chromatin-bound CtIP. Supplementary Fig. 4a shows that HORMAD1-depletion also led to reductions in chromatin-bound CtIP in other cell lines including A549 lung adenocarcinoma and MDA-MB436 TNBC cells.

CtIP-dependent DSB resection generates ssDNA which is rapidly bound by RPA. Therefore we determined the effect of HORMAD1 depletion on levels of RPA-containing nuclear foci in control and irradiated cells. As shown in Fig. 4d, the numbers of nuclei containing RPA IRIF were reduced in HORMAD1-depleted H1299 cells relative to control cultures, thereby recapitulating the effect of CtIP depletion. HORMAD1-depletion in A549 cells also led to a decrease in RPA-containing IRIF (Supplementary Fig. S4b). CtIP activity is regulated by the MRN complex (comprising MRE11, RAD50 and NBS1). In immunoblotting and confocal microscopy experiments, the chromatin binding of NBS1 was unaffected by HORMAD1-depletion (Fig. 4b, Supplementary Fig. S4c) indicating that the MRN complex is intact and relocalizes to DSB appropriately in the absence of HORMAD1. Taken together the results of Fig. 4 indicate that HORMAD1 facilitates the CtIP-mediated DSB resection phase of HR and stimulates production of the ssDNA substrate for strand invasion.

### Correlation between HORMAD1 mRNA expression and genome instability in lung cancer

Our molecular analyses (showing that HORMAD1 promotes HR in lung adenocarcinoma cell lines) mechanistically explain a recent report by Wang *et al*. showing that HORMAD1 confers resistance to PARP inhibitors^45^. However, both our findings and those of Wang *et al*. are inconsistent with a prior report suggesting that HORMAD1 inhibits HR in Triple Negative Breast Cancer (TNBC)^32^. Using a bioinformatics approach Watkins and colleagues identified a correlation between high HORMAD1 mRNA expression and copy number alterations (CNA) in TNBC^32^. Those workers attributed the increased CNA of HORMAD1-overexpressing breast cancer cells to reduced HR, and compensatory increases in alternative error-prone DSB repair activities.

**Figure 5:**
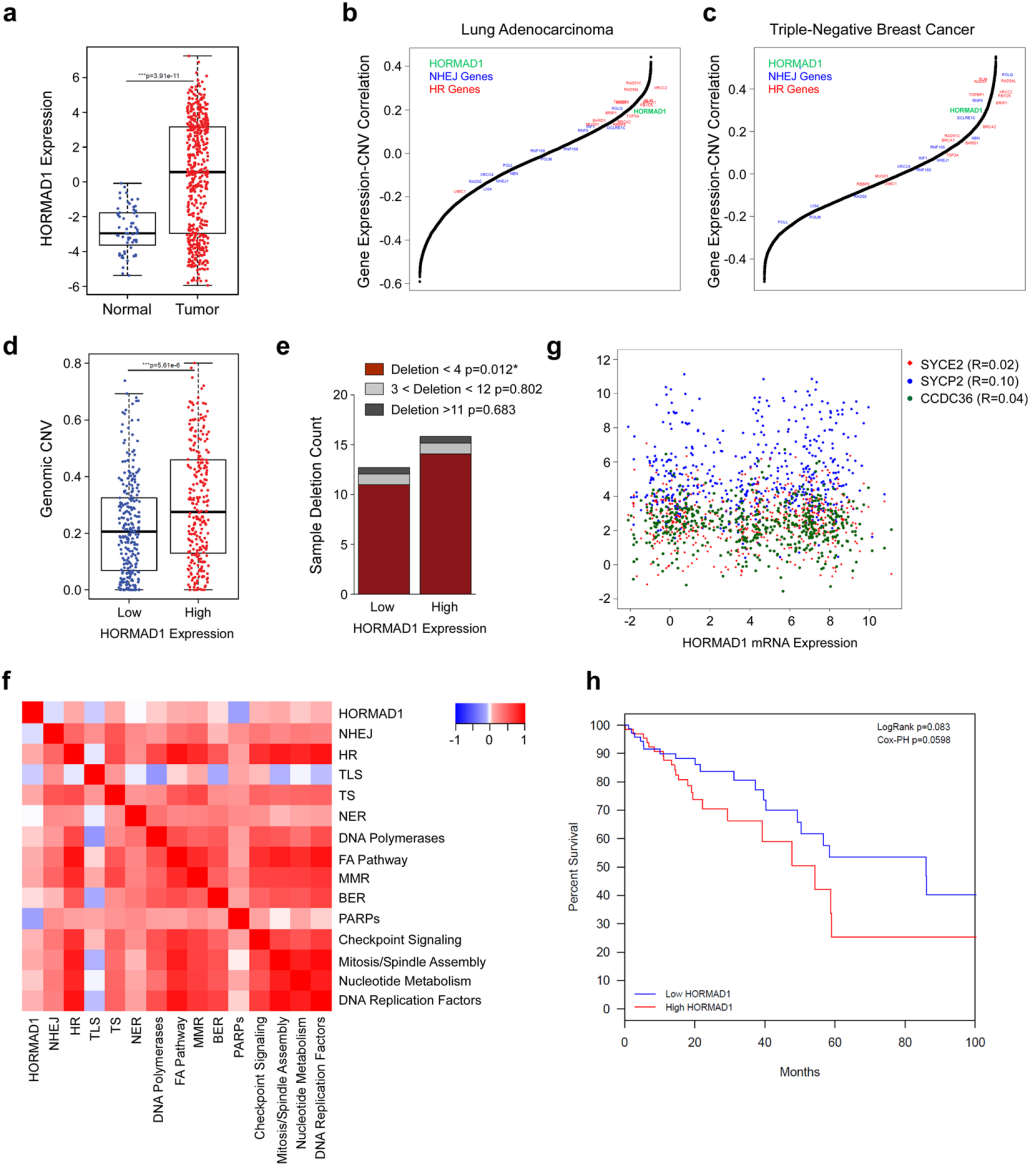
Genomic Correlates of HORMAD1 Expression in the TCGA Lung Adenocarcinoma (LUAD) dataset. **(a)** Boxplot of normalized HORMAD1 RNA expression in tumor and tumor-adjacent normal tissue samples in the LUAD samples. **(b**,**c)** Correlation between RNA expression and genomic copy number variation across all expressed. genes in LUAD and breast cancer samples respectively. HORMAD1 is indicated in green, other known HR genes are indicated in red, and NHEJ genes are indicated in blue. **(d)** Boxplot of genomic copy number variations by high vs low HORMAD1 expression. **(e)** Barplot of deletion frequency and length by high vs low HORMAD1 expression. **(f)** Heatmap of correlations between HORMAD1 and gene signature scores across DNA repair pathway gene set. **(g)** Plot showing correlation of HORMAD1 RNA expression and other CTAs in the LUAD dataset, showing no significant correlation between their expressions. **(h)** Kaplan-Meier plot of LUAD patients stratified by HORMAD1 expression.

Therefore, we sought to explain the discrepancy between our study and the report by Watkins *et al*. Our analysis of gene expression data from TCGA showed that similar to TNBC, HORMAD1 RNA expression was significantly upregulated in lung adenocarcinoma tumors relative to tumor adjacent normal tissue (Mann-Whitney p = 3.91e-11, Fig. 5a). This indicates that HORMAD1 activity is broadly upregulated in tumor cells. We asked whether HORMAD1 expression is associated with CNV (as defined using the same criteria as Watkins *et al*.^32^) in lung tumors. To do so we correlated the RNA expression of each gene in the transcriptome with the fraction of the genome that has undergone copy number variation in both the TCGA Lung Adenocarcinoma and Triple-Negative Breast Cancer datasets. We found that HORMAD1 expression, along with several HR-associated genes, is positively correlated with genomic copy number variation (Fig. 5b,c), indicating that HORMAD1 expression correlates with increased genomic instability. This was reinforced by our finding that tumor samples with high HORMAD1 expression have significantly higher levels of genomic instability (as measured by genomic copy number variation) than tumors with low expression of HORMAD1 (Fig. 5d, p = 5.61e-6). To validate our methodology, we correlated the expression of genes identified by Watkins *et al*. as defining the HiAiCNA, HiCnLOH, and LoSCINS groups with our copy number variation metric. We found that expression of the majority of HiAiCNA and HiCnLOH genes positively correlated with higher copy number variation, while LoSCINS correlated with higher genomic stability (Supplementary Fig. S5a,b). This was broadly consistent with the findings of Watkins *et al*. Furthermore, while there was no significant difference in longer SNP deletions, high HORMAD1 samples had significantly higher counts of <4 nucleotide deletions (Fig. 5e, p = 0.012). Therefore, consistent with the results of Watkins *et al*., high HORMAD1 expression is associated with CNV in lung adenocarcinoma and TNBC. We conclude that high HORMAD1 levels are correlated with genomic instability.

However, we noticed that the expression of many core HR pathway genes (including *BRCA1*, *BRCA2*, *BARD1*, *BRIP1*, *ABRAXAS1*, *UIMC1*, *RAD51*, *RAD51B*, *RAD51C*, *RAD51D*, *XRCC2*, *RAD54*) was strongly correlated with CNV, both in lung adenocarcinoma (Fig. 5b) and in TNBC (Fig. 5c). We further examined correlations between expression of HORMAD1 and genes representing core components of other major DNA repair pathways. As shown in Fig. 5f, Supplementary Fig. S6 and Supplementary Table 1, HORMAD1 expression was positively correlated with the expression of three DNA repair pathways involving a strand invasion step – namely HR (p = 0.029), Template Switching (p = 0.023) and the FA pathway (p = 0.048). HORMAD1 expression was not correlated with with other repair processes including NHEJ (p = 0.41) Trans-Lesion Synthesis (p = 0.21), and Nucleotide Excision Repair (p = 0.92). HR is a major source of mutational scars in cancer^46–49^. The CNV patterns examined by Watkins *et al*. which correlate with expression of HORMAD1 and other HR genes most probably represent an HR mutational signature. Expression of HORMAD1 in lung adenocarcinomas did not correlate with other expressed CT antigens that cooperate with HORMAD1 during meiotic recombination (Fig. 5g). The lack of a transcriptomic correlation between HORMAD1 expression and the expression of other CT antigens in patient tumors indicates that HORMAD1 signaling in these tumors is independent of other CT antigen functions. Therefore, putative effects of HORMAD1 on genome stability in cancer are likely to result from neomorphic activities unrelated to its HORMAD2 and CCDC36-mediated roles in germ cell development.

To further exclude the possibility that HORMAD1 is an inhibitor of HR we directly tested the effects of HORMAD1 overexpression on HR reporter gene activity and on DNA damage signaling. As shown by the DR-GFP reporter assays in Supplementary Fig. S7, HORMAD1 expression did not affect the HR-mediated repair of targeted DSB. Moreover, HORMAD1 overexpression in HORMAD1-deficient cells did not affect the magnitude or kinetics of the DNA damage response to IR. Therefore, we conclude that HORMAD1 does not inhibit HR.

Owing to the new role we defined for HORMAD1 in promoting HR, it was of interest to determine whether HORMAD1 expression was associated with particular oncogenic drivers and tumorigenic events in lung adenocarcinoma. As shown in Supplementary Fig. S8 & Supplementary Table 2, genomic alterations and gene expression events in HORMAD1 correlate with a diverse set of tumorigenic mechanisms in lung adenocarcinoma. Of note is the significant mutual co-occurrence of alterations with *DDR2*, encoding Dicoidin Domain Receptor 2, most likely because both genes residing on the chromosome 1q23.3 locus. As a result, HORMAD1-mediated genomic instability is likely to impact responsiveness to therapy in DDR2-driven tumors. We examined whether HORMAD1 expression levels were associated with patient survival in the LUAD cohort (Fig. 5H). While increased HORMAD1 expression adversely correlated with patient survival (HR = 1.1), this correlation did not reach the threshold of clinical significance (95% CI 0.996–1.213, p = 0.059). However, the majority of the patients in the cohort did not undergo postoperative radiotherapy. Therefore, it would be of interest to see if in larger cohorts of radiotherapy-treated patients, the clinical difference in response between HORMAD1-expressing and non-expressing tumors would reach the threshold of significance.

## Discussion

The new role we define for HORMAD1 in promoting DSB repair in cancer cells differs from how Hormad1 impacts repair radiation-induced DSB in mouse meiotic cells^50^. Carofiglio and colleagues examined repair kinetics of IR-induced DSB in *Spo11/Hormad1* double knockout mice^50^. Those workers observed fewer DSB repair foci remaining 24 h and 48 h after irradiation in *Spo11*^−/−^*/Hormad*^−/−^ double knockout spermatocytes, when compared to *Spo11*^−/−^ spermatocytes indicating that Hormad1 inhibits repair of exogenous DSBs in meiocytes^50^. Therefore, HORMAD1 promotes DSB repair in lung cancer cells but suppresses DSB repair in the germ line. However, the process of HR is fundamentally different in cells undergoing meiotic and mitotic divisions. DSB repair during meiosis occurs via inter-homolog recombination. In contrast, in mitotic cells (including neoplastic cells) recombination between homologues is suppressed and instead occurs between sister chromatids^51–53^, perhaps explaining how HORMAD1 can inhibit or promote DSB repair in different biological settings. Taken together, our work indicates that HORMAD1 acquires cancer cell-specific DSB repair functions that are independent of its roles in meiosis. This neomorphic role of HORMAD1 in HR is analogous to the cancer cell specific role of another CT antigen, MAGE-A4 which also activates an S-phase-coupled DNA repair mechanism to confer DNA damage tolerance^12,41^.

Our conclusion that HORMAD1 promotes DSB repair via HR mechanistically explains a recent study by Wang and colleagues showing that HORMAD1 promotes resistance to PARP inhibitors in basal-type breast cancers^45^. However, our findings and the work of Wang *et al*.^45^ apparently contrast with a prior report suggesting that HORMAD1 inhibits HR in breast cancer^32^.

The conclusions that HORMAD1 stimulates HR in two studies (our results and Wang *et al*.^45^) yet inhibits HR in other settings (Watkins *et al*.^32^) might be explained if HORMAD1 has opposing effects on HR in different cancers. For example, tissue-specific expression of putative HR pathway regulators targeted by HORMAD1 might explain why HORMAD1 stimulates HR in lung adenocarcinomas and basal breast cancer cells and but inhibits HR in TNBC. However, we have not observed inhibition of HR by HORMAD1 in any biological setting, including TNBC. Instead, based on results presented here and in Wang *et al*.^45^ we favor the idea that HORMAD1 generally promotes HR. Moreover, we suggest that some of the discrepancies between our work and the report by Watkins *et al*.^32^ are easily reconciled, as described below.

Watkins and colleagues identified HORMAD1 as a gene whose expression was associated with allelic-imbalanced copy-number aberrations (AiCNA) in a cohort of 126 Triple Negative Breast Cancers (TNBC)^32^. Fully consistent with Watkins and colleagues, our bioinformatic analyses of lung adenocarcinoma (TCGA) show that HORMAD1 expression is well correlated with AiCNA (Fig. 5, Supplementary Fig. S5). However, we noticed that AiCNA are not generally correlated with loss of HR genes (Fig. 5). Therefore, it is necessary to reevaluate the premise that the genomic instability associated with HORMAD1 expression in breast cancer (Watkins) and lung adenocarcinoma (this report) is necessarily caused by HR defects. Decomposition of genome sequence data into signatures is often useful for interpreting mutagenic processes. However, such analyses can be limited by the heuristic nature of associations between mutational patterns and molecular mechanisms – which often reflect opinions of researchers providing mutational signature annotations. To illustrate the heuristic nature of annotating mutation signatures, the forms of genome instability that Watkins and colleagues attribute to HR defects have also been suggested by others to result from excessive error-prone HR^48^. HR can be highly error-prone and drives both genome instability and cancer^46–49^. Approximately 25% of the genome contains repetitive sequences (including SINES, LINES, and microsatellites) and many genes are highly homologous or products of duplication events. Clearly HR events between dispersed homologous sequences can result in the deletions, duplications and translocations that are commonly observed in cancer^46,47^.

In cell culture models, RAD51 overexpression stimulates HR and induces chromosomal instability^48,54,55^. Expression of HR genes (including *RAD51*) is often elevated in cancer and associated with increased mutation rates^56^. Importantly, ablating RAD51 expression reduces genetic change^56^, directly demonstrating that HR is not error-free in carcinogenesis. Interestingly, although *BRCA1* mutant breast cancers are often categorized as HR-deficient, at least one BRCA1 mutation can lead to genetic instability via excessive resection of DNA breaks and hyper-recombination^57^. Clearly the prevalent view that HR is an error-free DNA repair mechanism is overly simplistic. Instead, HR should be viewed as a double-edged sword that can be error-free or produce rearrangements, and loss/gain of genetic information depending on context. This description of HR bears strikingly similarities to another S-phase-coupled genome maintenance mechanism – TLS, which can replicate damaged DNA in an error-prone or error-free, and is also activated by a CT antigen^12,41^.

Although our bioinformatic analyses of HORMAD1 expression in relation to mutation patterns are consistent with those of Watkins *et al*., the results of our DNA repair pathway studies diverge. For example, Watkins *et al*. did not detect redistribution of HORMAD1 to DSB. Retention of DNA repair factors at damaged DNA can be highly idiosyncratic and dependent on nuclear fixation and antibody staining conditions, possibly explaining why Watkins and colleagues did not detect HORMAD1 IRIF.

Another possibility is that HORMAD1 expression constructs used in different studies might differentially impact HR. For example the length or sequence of linkers that join proteins to epitope tags can have a major impact on DNA replication and repair factors^58^. In this regard, we note that our ectopically-expressed HORMAD1 redistributed to DSB and properly reconstituted the CtIP defect of HORMAD1-depleted cells. In our experiments these HORMAD1 activities required a specific C-terminal domain that is also important for developmental roles of HORMAD1 in meiosis. Therefore we have demonstrated functionality of our ectopically-expressed HORMAD1 and have provided a structural basis for HORMAD1 activities in the HR pathway. In contrast, Watkins *et al*. did not observe HORMAD1 foci or demonstrate rescue of their phenotypes with ectopically-expressed HORMAD1. It is possible that HORMAD1 constructs used by Watkins *et al*. were inactive or had dominant-negative effects that interfere with normal DSB repair processes. Alternatively, differences in culture conditions and cell cycle state could potentially explain discrepancies between our findings and those of Watkins *et al*.

Moreover, Watkins and co-workers did not determine phenotypic consequences of HORMAD1-deficiency in isogenic cells, and instead compared radiation-sensitivities of independent cell lines that were ranked solely based on HORMAD1 expression levels^32^. In studies with isogenic cells we observe HORMAD1-dependent HR pathway activation in several cell lines including lung adenocarcinoma and TNBC. Taken together, the most reasonable unifying interpretation of the collective data presented here and in reports by Watkins *et al*.^32^ and Wang *et al*.^45^ is that HORMAD1 promotes HR.

Interesting questions remain concerning the mechanisms of HORMAD1 expression in tumors, and the molecular basis for HORMAD1-induced HR in cancer cells. Similar to other CT antigens, HORMAD1 is likely to be de-repressed via epigenetic mechanisms^45^. However, in our analysis of TCGA data we also noticed that *HORMAD1* is often co-expressed with *DDR2* (encoding a collagen-binding receptor tyrosine kinase), a known oncogenic driver in NSCLC and other cancers. Interestingly, *HORMAD1* resides on the same chromosome as *DDR2*. Therefore the selective pressures for DDR2 amplification during carcinogenesis likely lead to co-amplification of *HORMAD1* gene whose expression likely helps define the biology of DDR2-driven cancers.

**Figure 6:**
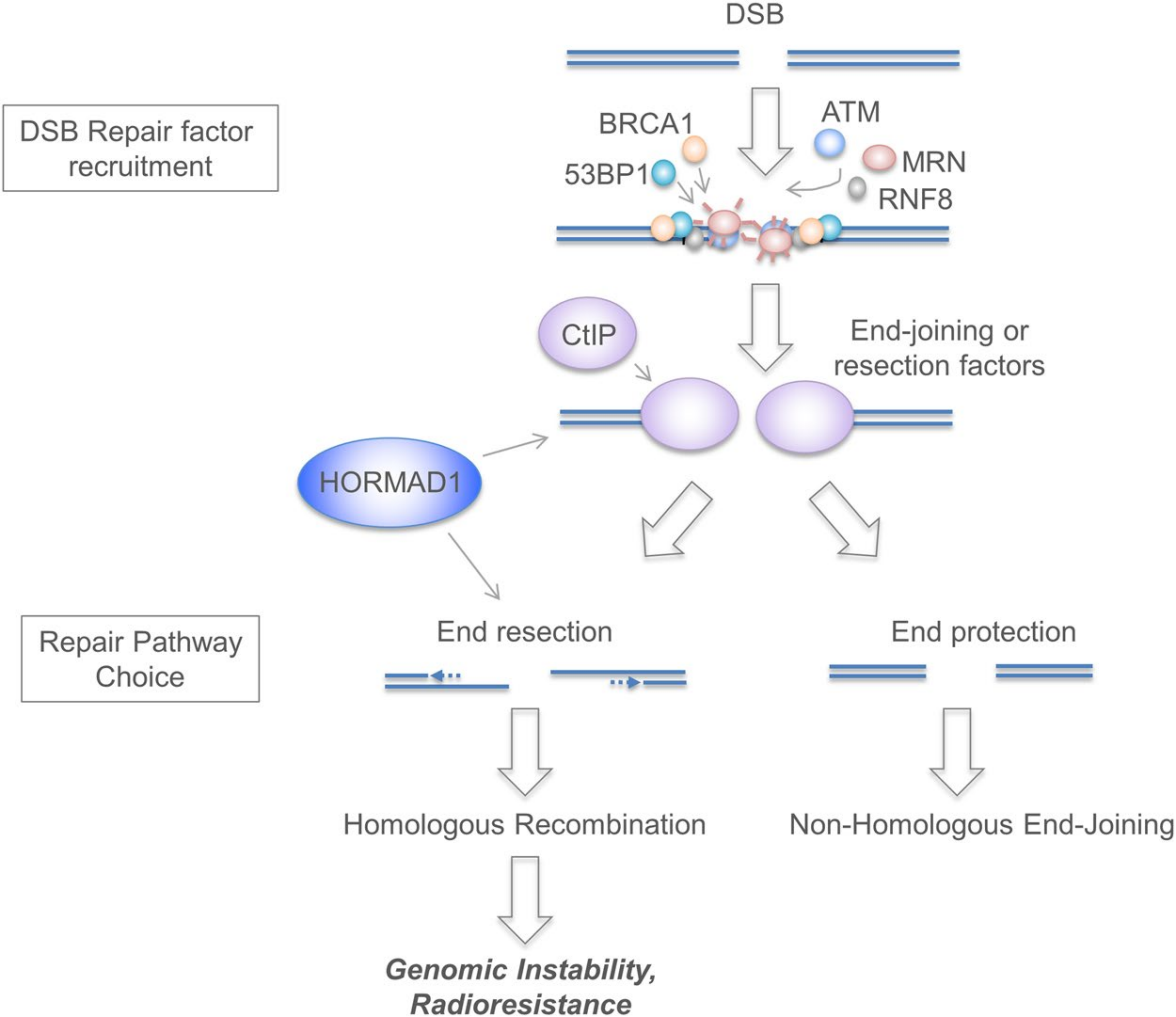
Scheme depicting role of HORMAD1 in DSB signaling and repair. Our immunofluorescence microscopy analyses (Fig. 4) show that formation of CtIP, RAD51 and RPA p34-containing IRIF is reduced in HORMAD1-depleted cancer cells (whereas 53BP1 and p95/NBS1 foci remain intact in the absence of HORMAD1). We infer that HORMAD1 contributes to HR pathway activation at (or prior to) the CtIP-mediated resection step, but does not affect the proximal stages of DSB signaling. HORMAD1-depletion leads to attenuation of DR-GFP/HR reporter activity (Fig. 3), prolonged IR-induced ATM signaling (Fig. 4), and radiosensitivity (Fig. 3), further demonstrating that HORMAD1 promotes the HR pathway.

Our results indicate that HORMAD1 facilitates a step distal to the initial recruitment of damage sensors such as ATM, the MRN complex and RNF8, but proximal to DSB resection (Fig. 6). We are considering several (non-mutually exclusive) molecular mechanisms for HORMAD1-mediated HR:

(i) *Interactions with other HORMA domain proteins*. By analogy with the MAGE-A4/RAD18 interaction that activates TLS, it is possible that HORMAD1 fortuitously associates with DNA repair protein(s) in a manner that stimulates HR. HORMAD1 contains a ~200AA HORMA domain found in diverse proteins (including Hop1, REV7 and MAD2) that participate in signal transduction through regulated assembly and disassembly of various protein complexes^22,23^. Meiotic HORMADs bind short closure motifs in their own disordered c-termini to mediate self-assembly into oligomers^44^. Our mutational analyses show that both the HORMA domain and the C-terminal peptide of HORMAD1 are critical for partitioning to IRIF. Therefore, we hypothesize that the HORMA domain of HORMAD1 mediates reversible binding to DNA repair proteins via a safety belt-based mechanism and/or that the C-terminal of HORMAD1 associates with another HORMA domain-containing protein to facilitate DNA repair in cancer cells. REV7 has a HORMAD1/2-like HORMA domain, resides at IRIF, inhibits DSB end resection and promotes NHEJ^59,60^. Interference with REV7 function by HORMAD1 could explain many of our results.
(ii) *HORMAD1 as a histone reader*. In its closed state, the HORMA domain of MAD2 is able to bind K3-methylated histone H3^61^. Therefore, HORMAD1 might read the histone code and participate in DNA repair via epigenetic mechanisms.
(iii) *Regulation of histone acetylation by HORMAD1. Drosophila* Hormad1 binds Msl1, a HAT-associated protein involved in DSB repair^62^. We have noticed that HORMAD1 depletion leads to reduced MSL1 levels and specifically decreases H4K16Ac levels (but not global Histone H4 acetylation in cancer cells (data not shown). H4K16Ac promotes HR^63–66^. Taken together, these results suggest the hypothesis that HORMAD1 interacts with MSL1 to stimulate H4K16 acetylation and promote a chromatin environment that is conducive to HR.

HORMAD1-dependent HR could endow neoplastic cells with a selective advantage, both as a driver of genetic change and as a mechanism of DNA damage tolerance. Cancer cells experience stressful environments and accumulate high levels of DNA damage from intrinsic sources including ROS and oncogene signaling^18,67,68^. DNA repair provides a potential means for neoplastic cells to tolerate intrinsic oncogene-induced stresses. Indeed, we recently showed that three major S-phase-coupled DNA repair mechanisms, namely TLS, HR, and alt-NHEJ allow cells to tolerate oncogene-induced replication stress^18^. Pathological HORMAD1-dependent HR may represent an important enabling characteristic of cancer cells.

Although cells expressing endogenous HORMAD1 clearly rely on this CTA to sustain HR, we have never observed stimulation of HR as a result of acute HORMAD1 expression. It is possible that in an HR-sufficient cancer cell, HORMAD1 overexpression does not lead to further increases in (already high) HR capacity. By analogy, overexpressing the CTA MAGEA4 in TLS-proficient cells also does not increase TLS activity^12^. However, in a recent study Wang *et al*. found that chronic HORMAD1 expression conferred resistance to PARP inhibitors in breast cancer xenografts (most likely due to increased HR)^45^. We favor the hypothesis that during the course of tumor development, cancer cells gradually develop dependency on neomorphic CTA functions to sustain tumorigenic activities such as genome maintenance^12,41^ and mitotic progression^69,70^, and resistance to apoptosis^8,71^.

Because normal cells lack CTA/HORMAD1-dependent DNA repair, the HORMAD1-HR signaling axis represents an appealing and therapeutically tractable molecular vulnerability. Targeted therapies against HORMAD1-mediated HR could sensitize cancer cells to intrinsic or radiation-induced DSB yet would be innocuous to normal somatic cells and devoid of side-effect toxicity. HORMAD1 is expressed at high levels in many cancers. It will be interesting to identify subsets of different cancers that depend most on HORMAD1 for DNA damage tolerance (perhaps including tumors driven by co-amplified *DDR2* oncogene). The biochemistry of many HORMA domain-containing proteins is now understood in considerable detail^22,23^. Harnessing this knowledge may enable development of chemical probes and small molecule inhibitors that disrupt interactions between specific HORMAD1 and its effectors to enhance radiotherapy and kill cancer cells.

## Materials and Methods

### Cell culture and transfection

H1299 and A549 lung carcinoma cells, Normal human Fibroblasts, HeLa cells, U2OS cells,MDA-MB436 cells, SUM159 cells and 293 T cells were from previously-specified sources^12,18^. All cell lines were cultured in Dulbecco’s modified Eagle medium (DMEM) supplemented with 10% fetal bovine serum (FBS) and penicillin–streptomycin. Plasmid DNA and siRNA oligonucleotides were transfected using Lipofectamine 2000 (Invitrogen) according to the manufacturer’s instructions with minor modifications as previously described^72^.

### Adenovirus construction and infection

Adenovirus construction, purification and infections were performed as described previously^35^. H1299 cells were typically infected with 0.1–1.0 × 10^9^ pfu/ml and titrated to achieve near-endogenous expression levels of Hormad1 and other proteins. NHF cells were infected with 2.0 × 10^9^ pfu/ml to achieve similar level expression as in H1299 cells.

### Expression plasmids

Mammalian expression pLX304 vectors encoding V5-HORMAD1, V5-HORMAD2, V5-SYCP1, V5-SYCE1, V5-CCDC36 and V5-SPO11 were purchased from the UNC Lenti-shRNA Core Facility and sequence-verified. HA-tagged HORMAD1 cDNA was generated by PCR-amplification using the pLX304 V5-HORMAD1 plasmid as a template. The resulting cDNA was subcloned into the pcDNA3.1(-) or pAC.CMV expression plasmids. HORMAD1 mutants harboring internal deletions and individual nucleotide substitutions were derived by PCR using conventional methods. All cDNA inserts were sequence-verified.

### RNA interference

siRNAs were incubated with Lipofectamine 2000 and serum-free Optimem for 15 minutes at room temperature. Cells were then trypsinized and resuspended in 1 ml of medium and plated directly into the siRNA/Optimem/Lipofectamine solution at 50% confluence and incubated for 48 hours. Sequences of siRNA oligonucleotides used here are as follows: Control non-targeting siRNA, 5′-UAGCGACUAAACACAUCAA-3′ (Dharmacon); HORMAD1 siRNA #1, 5′-GGA CAA AGA UGU AGA AGA U-3′, HORMAD1 siRNA #2, Dharmacon siGenome smart pool cat# M-018596-02; BRCA1, Dharmacon siGenome smart pool cat# M-003461-02; BRCH1, Dharmacon siGenome smart pool cat# M-010587-00; Lig4, Dharmacon siGenome smart pool cat# M-004254-00. For HORMAD1 depletions siRNA#1 was used unless otherwise indicated.

### Quantitative PCR to validate target depletion by siRNA

In some cases it was not possible to use immunoblot to validate knockdown of target proteins by siRNA due to lack of antibodies and quantitative PCR was used to validate siRNA-mediated knockdown of *CCDC36*, *BRCA1*, *LIG4*, *FANCJ* and *BRCA2* mRNAs. mRNA was extracted from siRNA-transfected cells using a RNeasy mini kit (Qiagen). cDNAs were generated using a SuperScript III First-Strand Synthesis System (Invitrogen). Quantitative PCR was performed using a Quantstudio 7 Flex Real-Time PCR System with SYBR green real-time PCR master mix (Applied Biosystems). The ΔΔCt method was applied to calculate relative mRNA levels. The quantitative PCR primer sequences used in these experiments were: CCDC36 F-GCCAGCAGCCAGAGGTATAA; R-ACTGGGAATCACTGAGACTGG

BRCA1: F-GAA ACC GTG CCA AAA GAC TTC; R-CCA AGG TTA GAG AGT TGG ACA C

LIG4: F-AGCAAAAGTGGCTTATACGGATG; R-TGAGTCCTACAGAAGGATCATGC;

BACH1: F-TCCAAGCACACCACCTTCTG; R-TGGATGCCTGTTTCTTAGCAG

BRCA2 F-GCCCCTTATCTTAGTGGGAGAAC; R-GTGCGAAAGGGTACACAGGT

*β*Actin: F-AGAAAATCTGGCACCACACC; R-AGAGGCGTACAGGGATAGCA.

### Genotoxin Treatment

For IR treatment, cells were irradiated with RS-2000 X-ray irradiator (Rad Source Technologies) maintained by the UNC Lineberger Cancer Center. For IRIF experiments, cells were irradiated with 8 Gy X-ray. For other experiments such as IR recovery western blots and clonogenic survival assays, cells were irradiated with dose indicated in each figure. For UVC treatment, growth medium was removed from cultured cells and replaced with PBS. The resulting culture dishes plates were irradiated using a UV-cross-linker (Stratagene), or left untreated for control. The UVC dose delivered to the cells was confirmed with a UV radiometer (UVP, Inc.). Following UV- or sham-irradiation cells were re-fed with complete growth medium and returned to the incubator. For CPT, ETO and olaparib treatments cells were treated with various doses of corresponding chemical as indicated in the main text.

### Fluorescence Microscopy

H1299 cells were grown to ~60% confluency on glass-bottom plates (Mat-tek) and then infected with with a HA-Hormad1 expression adenovirus. 24 h after infection cells were X-ray irradiated (8 Gy) or sham-treated and fixed 2 h later for staining with anti-HA and other antibodies then fixed-cell imaging on a Zeiss 700 confocal microscope, as described previously.2,4 All IF samples were pre-extracted with ice-cold cytoskeleton buffer (CSK buffer; 10 mM Pipes, pH 6.8, 100 mM NaCl, 300 mM sucrose, 3 mM MgCl_2_, 1 mM EGTA, 1 mM DTT, 0.1 mM ATP, 1 mM Na_3_VO_4_, 10 mM NaF, and 0.1% Triton X-100) for 5 mins on ice then fixed with 2% PFA for 15 mins at room temperature except for NBS1 staining. For NBS staining, cells were pre-extracted with CSK on ice for 5 mins, followed by incubation in cytoskeleton stripping buffer (10 mM Tris [pH 7.4], 10 mM NaCl, 3 mM MgCl_2_, 1% Tween 40, 0.5% sodium deoxycholate) for 5 min on ice as described previously^12^. Antibodies used were 53BP1 (1:400 sc22760 Santa Cruz); γH2AX (1:500 05-636 Millipore); HA tag (1:300 11867423001 ROCHE); MDC1 (1:300 A300-051A Bethyl); MYC tag (1:500 2276 Cell Signaling); NBS1 (1:500 NB100-143 Novus); RAD51 (1:500 sc8349 Santa Cruz); RPA2 (1:200 NA18 Millipore); V5 tag (1:300 ab9116 abcam). To enumerate DSB-induced foci, at least 100 nuclei were examined and scored as positive or negative for IRIF under each experimental condition.

### Quantification of Immuno-fluorescent signals Co-localization

The fluorescence intensities of HORMAD1 and γH2AX signals were plotted using the Carl Zeiss ZEN software. The Pearson’s correlation coefficient (PCC) between different immunofluorescent signals described by Dunn *et al*. was automatically calculated using IMARIS microscopy image analysis software (Version 9.0.2). A PCC value of 1 represents perfect co-localization and a PCC value of −1 represents complete mutual exclusivity.

### Immunoprecipitation and Immunoblotting

To prepare extracts containing soluble and chomatin-associated proteins, monolayers of cultured cells typically in 60 mm plates were washed three times in ice-cold PBS and lysed in 500 μl of ice-cold cytoskeleton buffer (CSK buffer; 10 mM Pipes, pH 6.8, 100 mM NaCl, 300 mM sucrose, 3 mM MgCl_2_, 1 mM EGTA, 1 mM DTT, 0.1 mM ATP, 1 mM Na_3_VO_4_, 10 mM NaF, and 0.1% Triton X-100) freshly supplemented with protease inhibitor cocktail and phostop (Roche). Lysates were centrifuged at 1,000 g for 2 min to remove the CSK-insoluble nuclei. Supernatants were removed and further centrifuged at 10,000 g for 10 min to obtain a clarified fraction containing a mixture of cytosolic plus nucleosolic proteins. The detergent-insoluble nuclear fractions were washed once with 1 ml of CSK buffer and then resuspended in a minimal volume of CSK prior to analysis by SDS-PAGE and immunoblotting.

For Immunoblotting, cell extracts or immunoprecipitates were separated by SDS-PAGE, followed by incubation overnight with the following primary antibodies: p-ATM (sc-47739), 53BP1 (sc-22760), RNF8 (sc-271462), Rad51 (sc-8349), GAPDH (sc-32233), all from Santa Cruz Biotech; NBS1 (A300-187A) from Bethyl Laboratories; MYC-Tag (2276), from Cell Signalling; γH2AX (05-636) from Millipore; ORC4 (H83120) from Transduction labs; β-Actin (A2228) from Sigma; CtIP (61141) from Active Motif; HA tag (11867423001) from ROCHE; HORMAD1 (ab178432) from abcam.

### Flow cytometry

For BrdU labeling experiments, cells were labeled with 10 μM BrdU immediately prior to harvest. Cells were collected by trypsinization, fixed in 35% ethanol for 24 h, then stained with anti-BrdU and propidium iodide as previously described^35^. Stained nuclei were analyzed by flow cytometry on an Accuri C6 flow cytometer (BD, Oxford, UK) using the manufacturer’s software.

### End-joining and homologous recombination reporter assays

H1299 cells were transfected with pimEJ5GFP^73^ or pDRGFP^74^ then selected in puromycin. Individual clones were picked for each reporter and used in the reporter assay. Reporter cells were transfected with corresponding siRNAs. 24 hours later, cells were infected with I-SceI expressing adenovirus. Cells were harvested 48 hours after adenovirus infection and analyzed by flow cytometry. GFP-positive cells were enumerated as described^73,74^.

### Bioinformatics

TCGA Lung Adenocarcinoma RNA Expression, mutation, genomic alteration, and clinical information were downloaded from the Broad Institute Firehose Pipeline (http://gdac.broadinstitute.org). RNA expression was downloaded in a normalized RSEM file. Expression values were log_2_ transformed, and genes that were expressed in less than 80% of all samples were filtered out. Missing values were imputed using K-nearest neighbor imputation. Gene signature z-scores were obtained by normalizing each gene within a signature across all samples in the dataset and taking the median of all normalized gene levels within a gene signature as the z score of the gene signature. A Pearson correlation was performed for all metrics in which a correlation metric is indicated. Mutation/indel data were derived from sample mutation data that were downloaded as.maf files. Deletions were thresholded at lengths of 3 and 11 bp to be consistent with previous work^75^. Survival status and overall survival were determined on the basis of the data provided. Oncoprint figures were produced using the downloaded TCGA mutation and copy number alteration (CNA) data. Genes were selected on the basis of previously being identified as having significant mutations or CNAs within the gene. Watkins *et al*. gene lists were obtained from their publication^32^.

### Reproducibility

All immunofluorescence microscopy and immunoblotting data shown are representative of results obtained in at least three independent experiments performed on separate occasions unless otherwise stated. In all flow cytometry experiments (for cell cycle and DNA repair reporter assays) duplicate samples were analyzed and the data shown are representative of experiments that were performed on at least three separate occasions.

### Statistics

All statistical analyses were performed using GraphPad Prism version 6.0 (GraphPad Inc., La Jolla, CA). Data were analyzed using the two-tailed unpaired student’s t-test or two way ANOVA with Tukey’s multiple comparison test as appropriate. p < 0.05 was considered statistically significant. * denotes p < 0.05, ** denotes p < 0.005, *** denotes p < 0.0005 and **** denotes p < 0.0001.

## Electronic supplementary material

Supplementary Figures

## Data Availability

All data generated or analyzed during this study are included in this published article (and its Supplementary Information files).
