# Supplementary material for: The Cancer/Testes (CT) Antigen HORMAD1 promotes Homologous Recombinational DNA Repair and Radioresistance in Lung adenocarcinoma cells
Source: Sci Rep. 2018 Oct 17;8:15304. doi: 10.1038/s41598-018-33601-w (PMC6192992; doi:10.1038/s41598-018-33601-w)
Supplement: Supplementary file 1 — Supplementary Figures [file 41598_2018_33601_MOESM1_ESM.pdf]

The Cancer/Testes (CT) Antigen HORMAD1 promotes Homologous Recombinational DNA Repair and Radioresistance in Lung adenocarcinoma cells

Yanzhe Gao[1], Jordan Kardos[2], Yang Yang[1], Tigist Y. Tamir[3], Elizabeth Mutter-Rottmayer[1], Bernard Weissman[1,2], Michael B. Major[3], William Kim[2] and Cyrus Vaziri[1,2]

[1]Department of Pathology and Laboratory Medicine, University of North Carolina at Chapel Hill, 614 Brinkhous-Bullitt Building, Chapel Hill, NC 27599, USA

[2]Lineberger Comprehensive Cancer Center, Curriculum in Genetics and Molecular Biology, and Department of Biochemistry and Biophysics, University of North Carolina, Chapel Hill, NC 27599, USA

[3]Department of Cell Biology and Physiology, Lineberger Comprehensive Cancer Center, University of North Carolina at Chapel Hill, Chapel Hill, NC 27599-7295

Correspondence and requests for materials should be addressed to cyrus_vaziri@med.unc.edu, Tel: 919-843-9639, Fax: 919-966-5046

Supplementary Figures

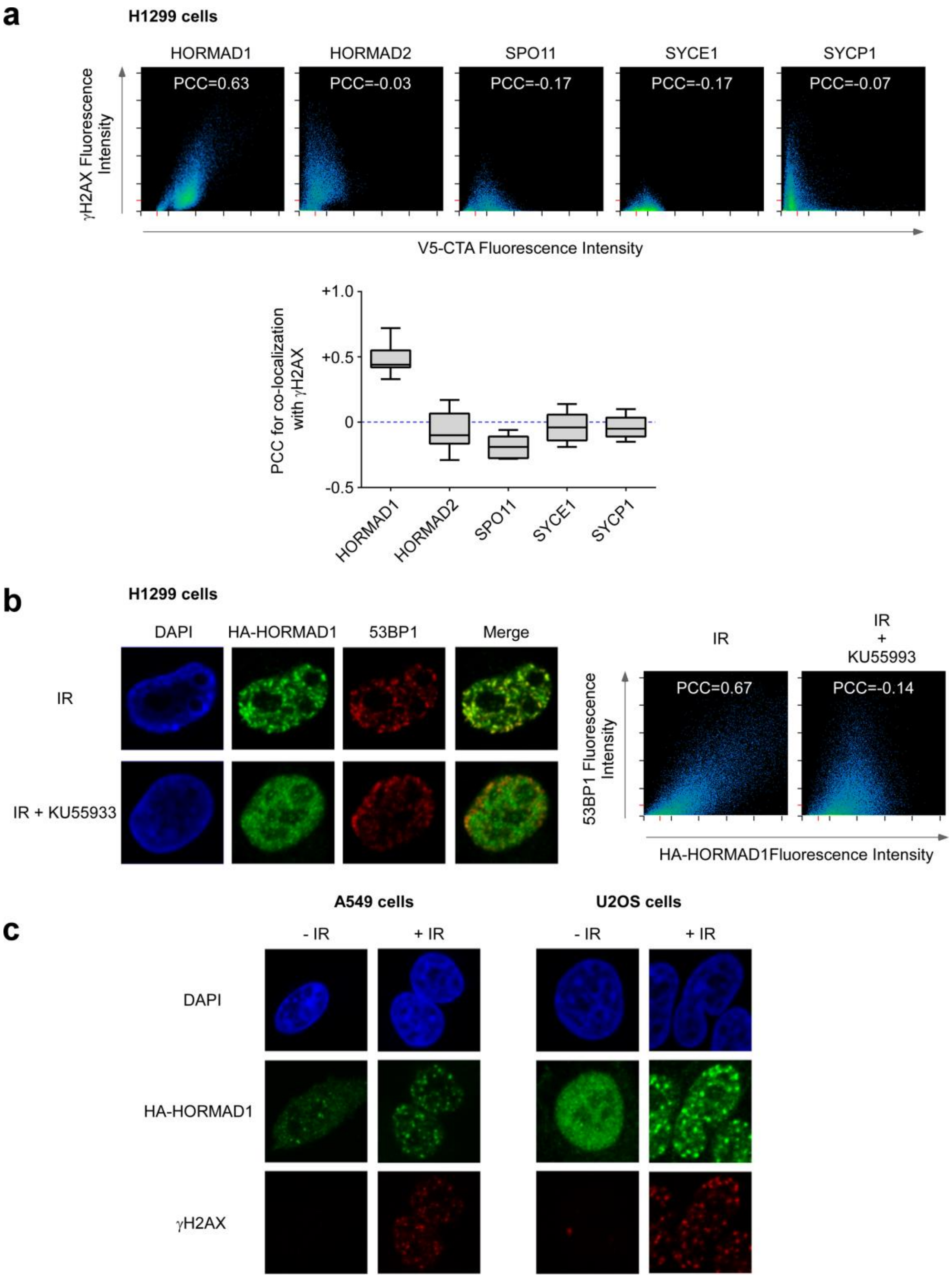

Supplementary Figure S1 (accompanies Fig. 1) Defining the effect of DNA damage on subcellular distribution of HORMAD1.

(a) Imaging analysis to empirically measure degree of co-localization between HORMAD1 and known DNA damage markers. The fluorescence intensities of HORMAD1 and γH2AX signals were plotted

using the Carl Zeiss ZEN software. The Pearson's correlation coefficient (PCC) for HORMAD1 localization in relation to γH2AX was calculated using IMARIS microscopy image analysis software. A PCC value of 1 represents perfect co-localization and a PCC value of -1 represents complete mutual exclusivity. Therefore, the PCC values indicate co-localization of γH2AX with HORMAD1 but not with the other CTAs tested. The histogram shows the mean PCC values obtained for CTA/γH2AX co-localization obtained in three independent analyses and the error bars represent the range.

(b) Effect of ATMi on co-localization between HORMAD1 and 53BP1. HA-HORMAD1 was expressed in H1299 cells using a recombinant adenovirus. 24 h post-infection, some cultures were treated with 10 μM KU55933 for 1 h. Control and KU55933-treated cells were conditionally irradiated (10 Gy) and 1 h later the subcellular distribution of HA-HORMAD1 in relation to 53BP1 was analyzed by confocal microscopy and PCCs were determined using IMARIS microscopy image analysis software. The PCC values indicate that the co-localization of HORMAD1 and 53BP1 (PCC=0.67) is impaired following ATM inhibition (PCC=0.14).

(c) HORMAD1 redistributes to IRIF in cancer cell lines. HA-HORMAD1 was expressed in replicate cultures of A549 lung adenocarcinoma and U2OS osteosarcoma cells using a recombinant adenovirus. 24 h post-infection, some cultures were irradiated (10 Gy) and 1 h later the subcellular distribution of HA-HORMAD1 in relation to γH2AX was analyzed by confocal microscopy.

	H522	H2228	H358	H1359	H1299
HORMAD1	0	0.442308	0.3062279	1	0.3314947
HORMAD2	0	0	1	0.988152	0
SYCP1	1	0	0	0	0
SYCE1	0.0029758	1	0.583978	0	0.7885649
SPO11	0	0	0	0	0
CCDC36	0.0015661	1	0.030507	0.0075364	0.7597417

Supplementary Figure S2 (accompanies Fig. 2) Heat map showing relative levels of HORMAD1 and other CT Antigen expression (obtained by RNASeq) from the indicated lung adenocarcinoma cell lines. mRNA expression levels are normalized to the highest expressing cell lines.

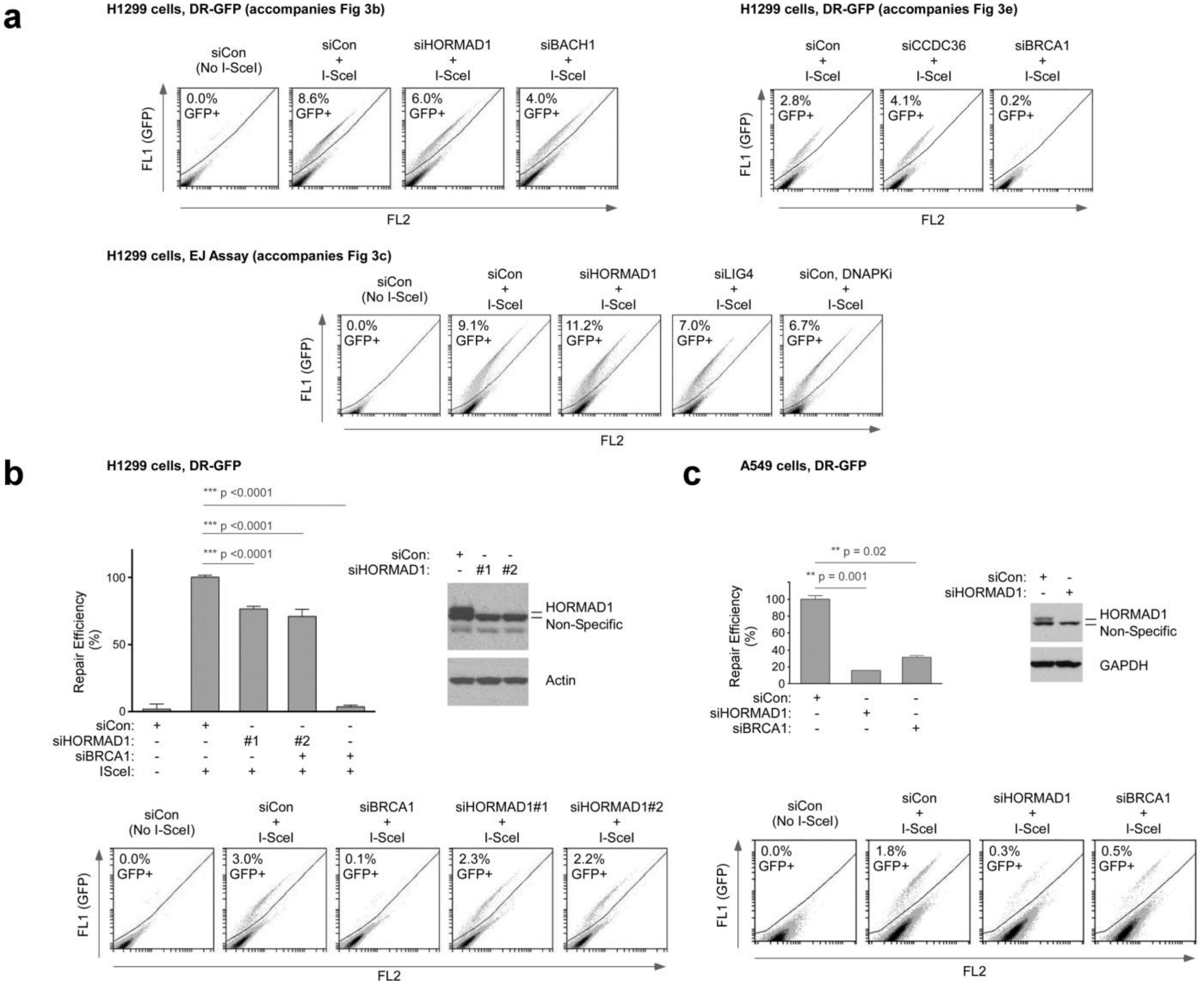

Supplementary Figure S3 (accompanies Fig. 3) HORMAD1 promotes HR in cancer cell lines.

(a) FACS profiles showing the percentage of GFP-positive cells generated during repair of I-SceI-induced DNA double strand breaks in the stably-integrated DR-GFP and EJ reporter plasmids. Each plot shows one representative FACS profile from each triplicate set of samples analyzed for each experimental condition in Fig. 3.

(b) Multiple HORMAD1 siRNAs attenuate HR activity in lung adenocarcinoma cells. Replicate plates of H1299 cells harboring the stably-integrated DR-GFP reporter construct were transfected with the indicated siRNAs (against HORMAD1, BRCA1, or non-targeting siRNA). 24 h later the siRNA-treated cells were transfected with an ISceI expression plasmid (to induce DSB in the DR-GFP locus) or with an empty control vector. After 24 h cells were trypsinized and GFP-expressing populations (resulting from HR-mediated reconstitution of a silent GFP allele) were enumerated by flow cytometry. Error bars indicate the standard error of the mean from three independent experiments.
The immunoblot shows relative of HORMAD1 expression in replicate plates of siRNA-transfected cells.

(c) Replicate plates of A549 cells harboring stably-integrated DR-GFP were transfected with the indicated siRNAs and tested for repair of ISceI-induced breaks as described for (A). Error bars indicate the standard error of the mean from three independent experiments.

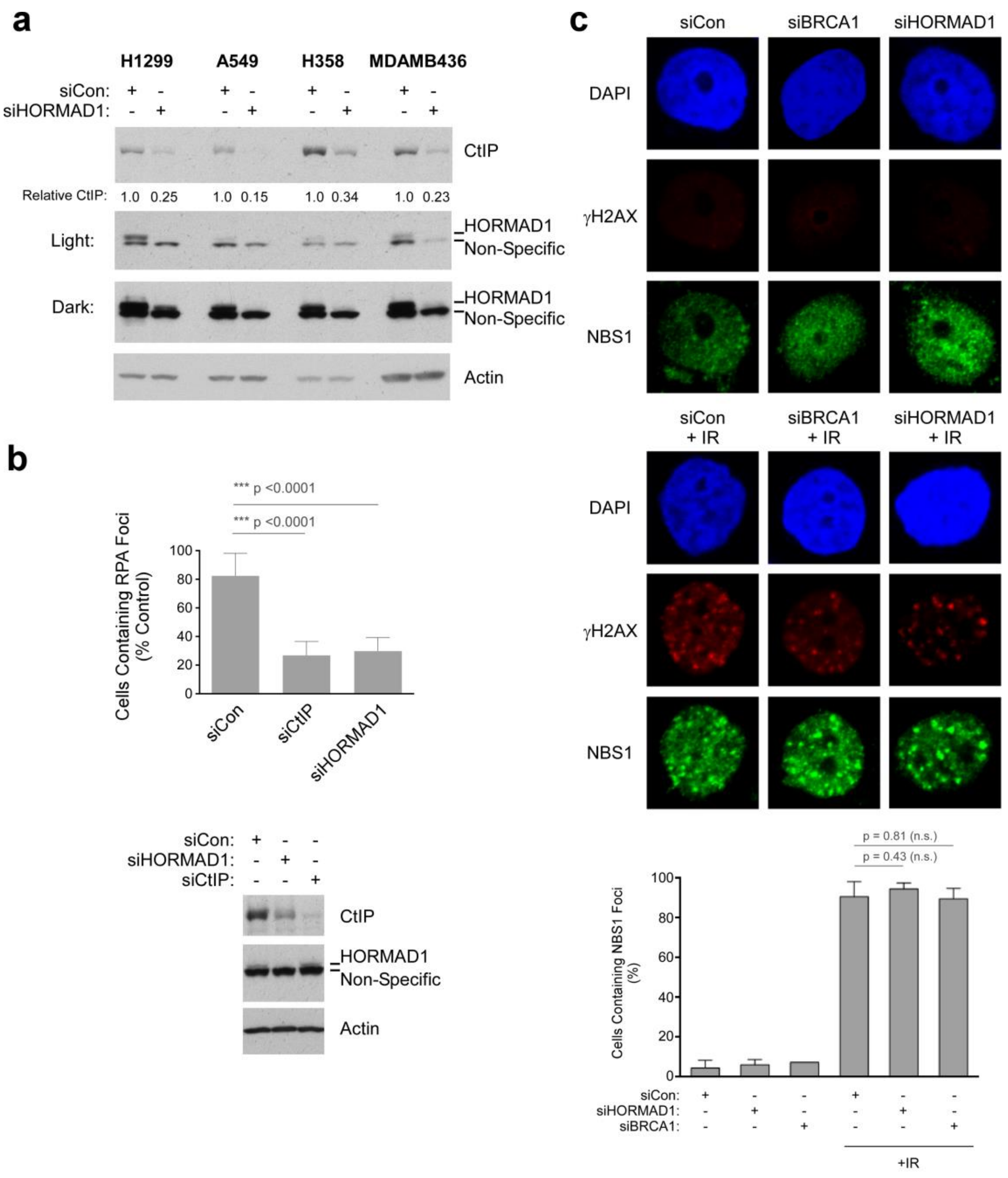

Supplementary Figure S4 (to accompany Fig. 4) HORMAD1 facilitates HR but not impact NHEJ

(a) HORMAD1 sustains chromatin-bound CtIP in cancer cell lines. Lung adenocarcinoma (H1299, A549, H358) and breast cancer (MDA-MB436) cell lines were transfected with siHORMAD1 or non-targeting control RNA (siCon). 48 h post-transfection cells were treated with IR (10 Gy) to induce DSB. Chromatin-bound proteins were analyzed by SDS-PAGE and immunoblotting with the indicated antibodies.

(b) HORMAD1 sustains RPA foci in A549 cells. A549 cells were transfected with siHORMAD1, siCtIP, or with non-targeting siCon RNA. 48 later cells were irradiated (10 Gy) and 2 h later nuclei were fixed and analyzed for RPA foci using immunofluorescence confocal microscopy. The percentage of cells showing RPA IRIF are quantified.

(c) HORMAD1-depletion does not affect redistribution of NBS to IRIF. H1299 cells were transfected with siCon, siBRCA1 or siHORMAD1 RNAs. 48 h post-transfection cells were irradiated (10 Gy) and 1 h later nuclei were analyzed for γH2AX and NBS1 foci using immunofluorescence confocal microscopy. 100 nuclei were analyzed for each experimental condition. The results of analyses are shown on the histogram and error bars indicate the standard deviation from three different experiments.

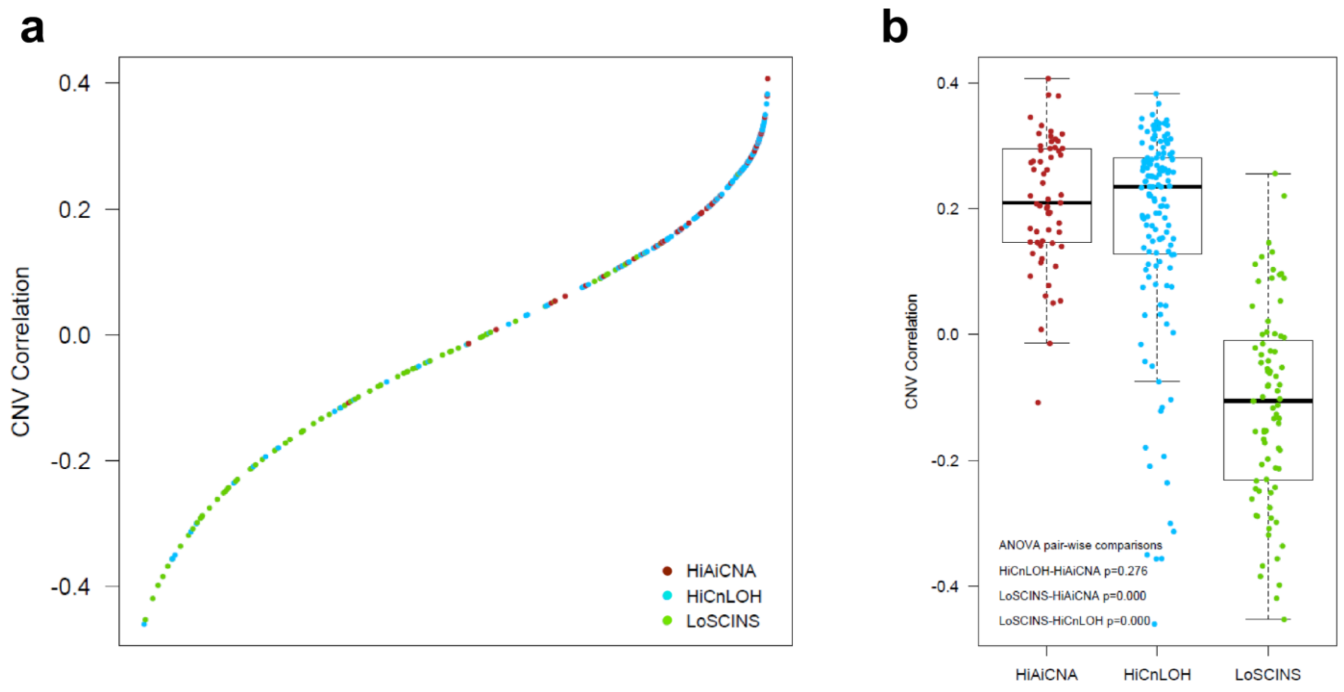

Supplementary Figure S5. Validation of Watkins et al subtypes and gene lists in lung adenocarcinoma

(a) Plot showing the correlation between genes defining Watkins et al subtypes and correlation to genomic copy number variation.

(b) Boxplot showing differences in correlation to copy number variation of genes associated with Watkins et al subtypes.

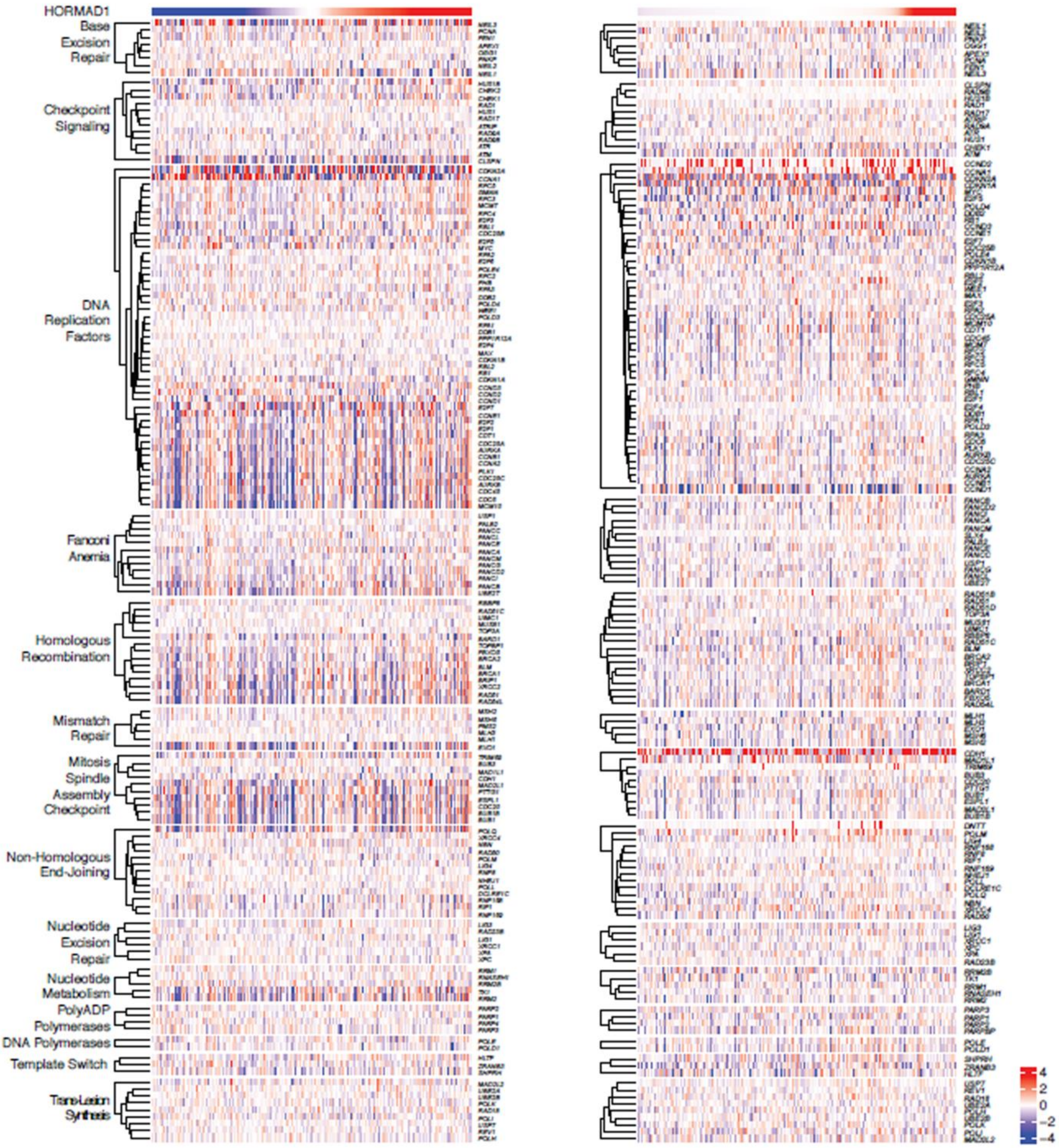

Supplementary Figure S6. Heatmap of individual DNA repair pathway expression in the LUAD dataset shows correlation to HORMAD1 expression. Heatmap of individual gene components of DNA repair pathways summarized in Fig 5F, supervised by increasing HORMAD1 expression.

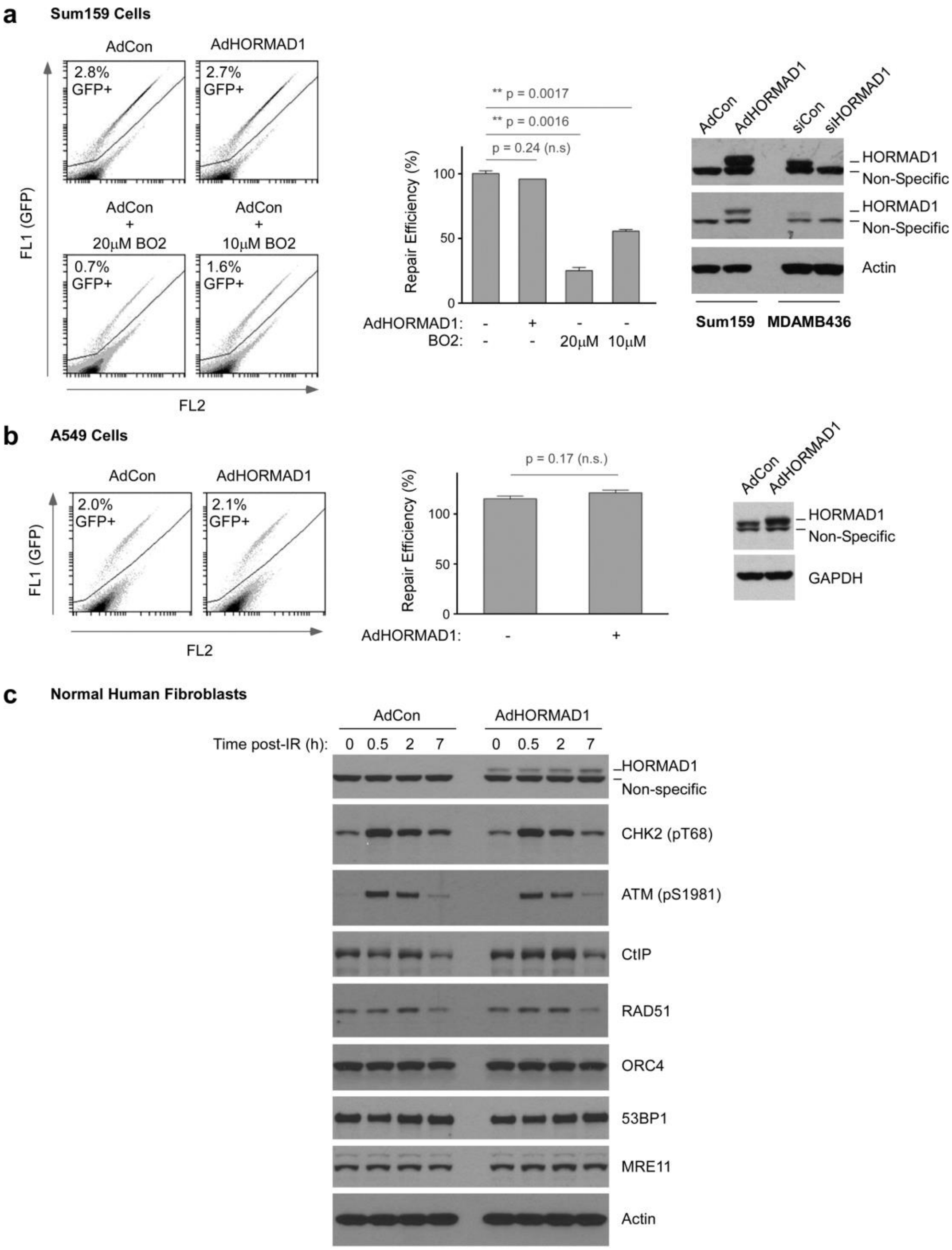

Supplementary Figure S7. HORMAD1 overexpression does not affect HR activity or the DNA damage response to IR.

(a)-(b) The DR-GFP plasmid was transfected into Sum159 or A549 cells and clones stably expressing the reporter were isolated. Cells were sequentially transduced with HORMAD1 adenovirus (or empty adenoviral vector vector for control) or were treated with the RAD51 inhibitor BO2 for 24 hr prior to

inducing DSB with ISceI. Levels of ectopically-expressed HORMAD1 in the transduced cells are indicated in the immunoblots. As shown in panel (a), the levels of HORMAD1 attained by adenoviral expression in SUM159 cells (which lack endogenous HORMAD1) were comparable to HORMAD1 levels in the representative HORMAD1+ TNBC line MDA-MB436. Representative flow profiles showing GFP+ cells are shown for one of the experiments. The histograms show a summary of three independent experiments with duplicate determinations and error bars indicate the standard error of the means. (Please note that some of the error bars in the panels are very small and cannot be seen at the final resolution)

(c) NHF cells were infected with AdHORMAD1 or a control 'empty' adenoviral vector ($3x10^{10}$/ml). 48 h later cells were treated with IR (10 Gy) and extracts were prepared at the indicated times after irradiation. Protein samples were analyzed by SDS-PAGE and immunoblotting with antibodies against the indicated proteins.

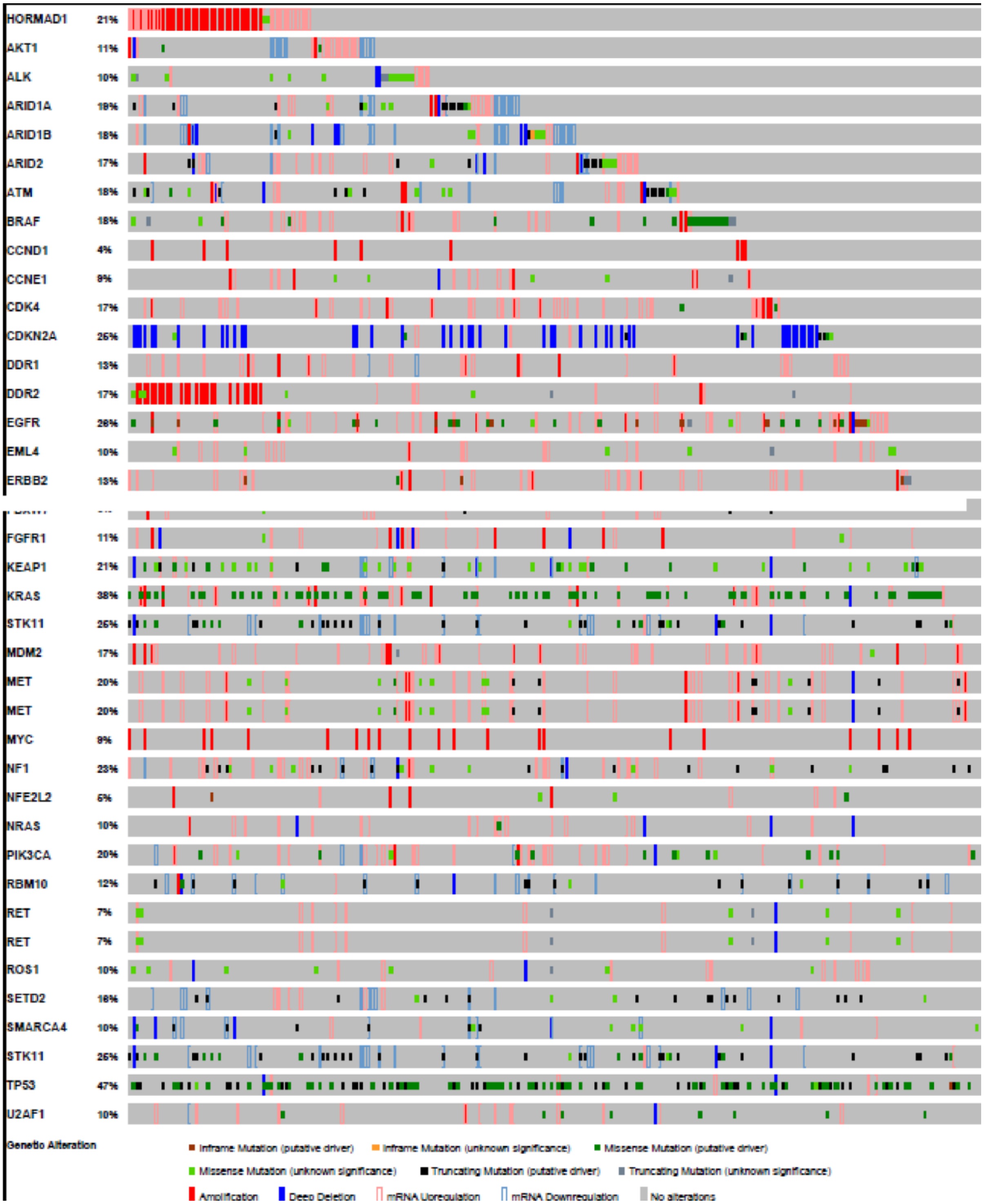

Supplementary Figure S8. Oncoprint showing mutations and copy number alterations in HORMAD1 and other significantly mutated genes in Lung Adenocarcinoma
The figure was generated through the cBioPortal oncoprint interface to visually show co - occurrence/exclusivity of alterations in HORMAD1 with LUAD SMGs.

Fig 2B

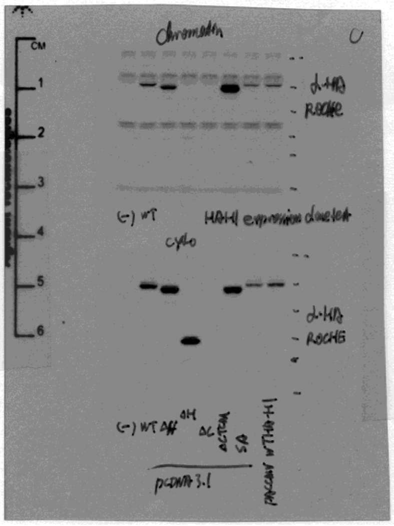

Fig 2E

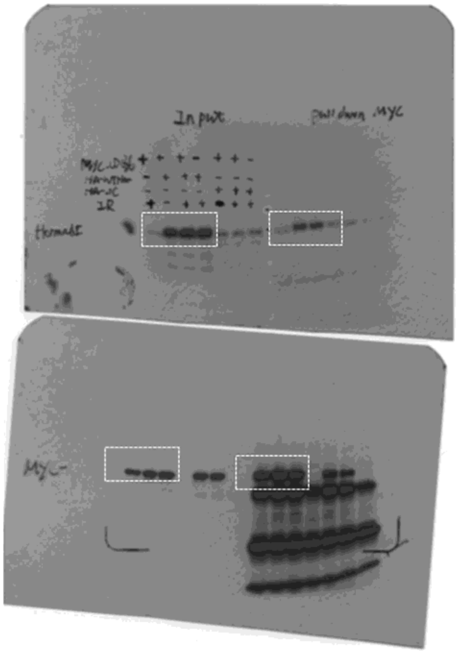

Fig 3A

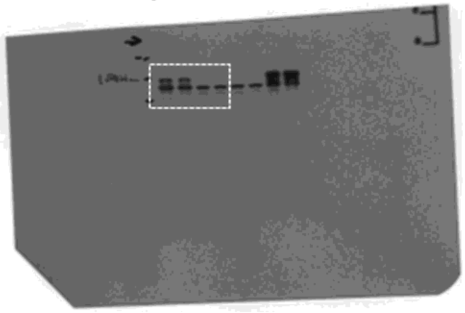

Fig 3E

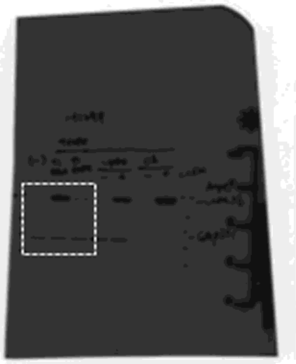

Supplementary Fig. 9 uncropped blots

Figure 1B

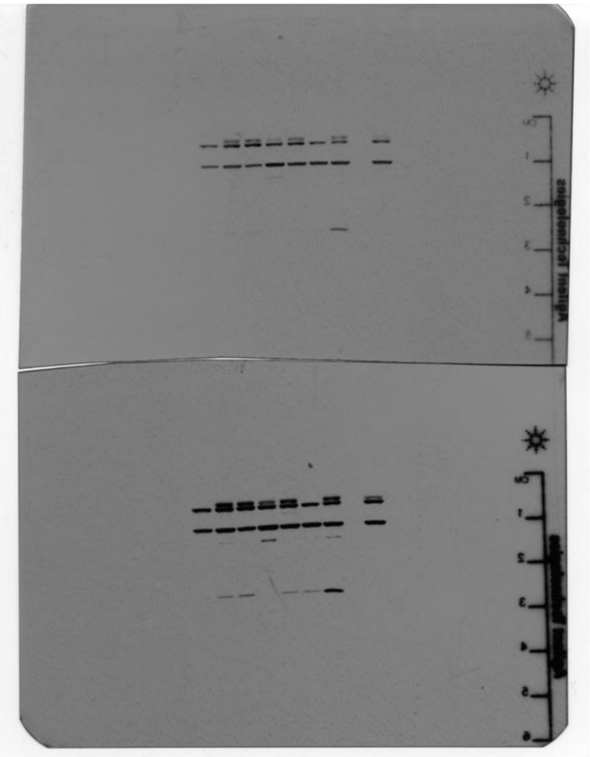

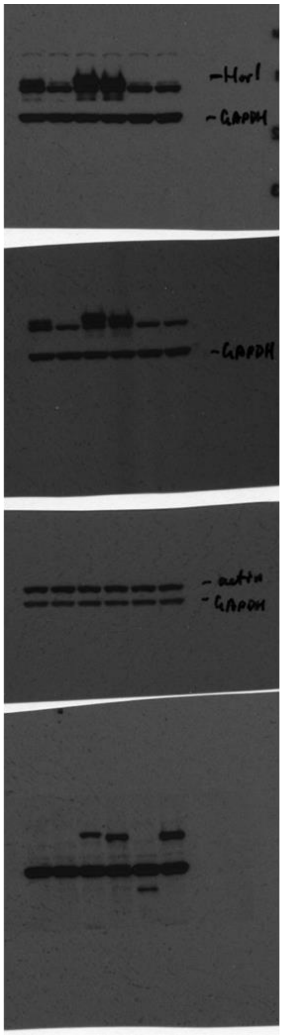

Figure 4C

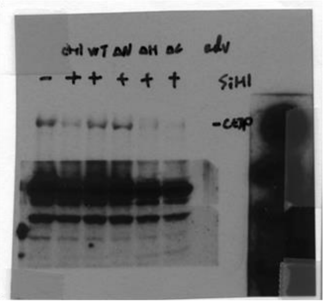

Supplementary Fig. 9 continued uncropped blots

Fig S7C

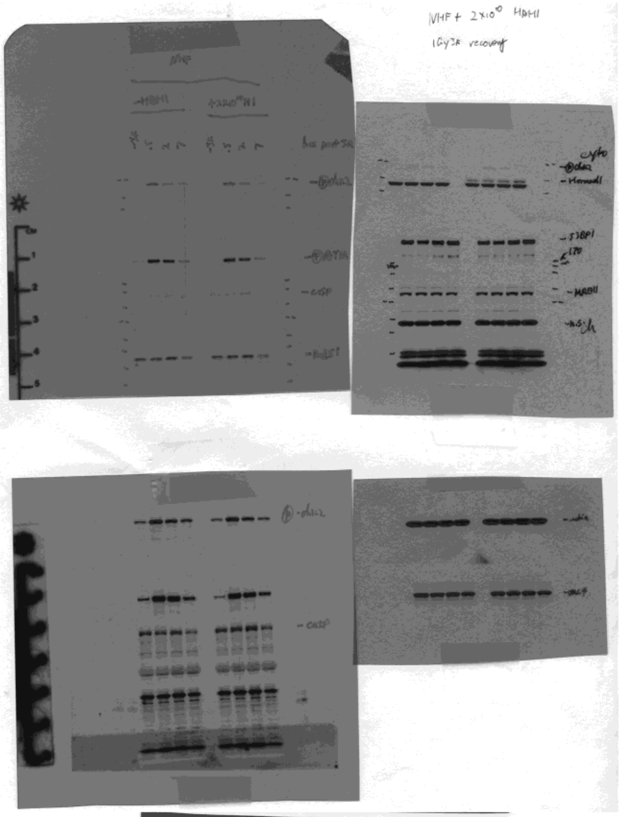

Fig 4B

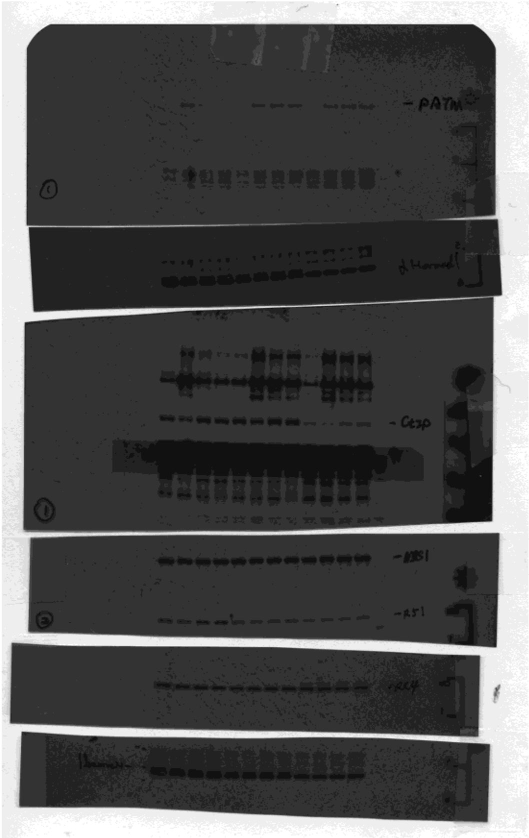

Fig S3A

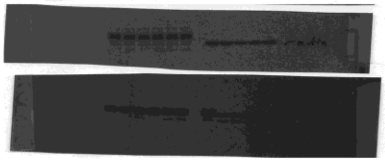

Supplementary Fig. 9 continued uncropped blots

	HORMAD1_Correlation	Significance_pvalue
HORMAD1	1	0
Non_Homologous_End_Joining	-0.036644555	0.4057
Homologous_Recombination	0.095465288	0.02998
Trans_Lesion_Synthesis	-0.055206812	0.2101
Template_Switch	0.100073955	0.02287
Nucleotide_Excision_Repair	-0.00390711	0.9294
Repair_Replication_DNA_Polymerases	0.054211626	0.2185
Fanconi_Anemia	0.086720149	0.04875
Mismatch_Repair	0.110596331	0.01186
Base_Excision_Repair	0.038660894	0.3803
PolyADP_Ribose_Polymerases	-0.14750096	0.0007679
Checkpoint_Signaling	0.080151414	0.06861
Mitosis_Spindle_Assembly_Checkpoint	0.097134111	0.02721
Nucleotide_Metabolism	0.057311255	0.1932
DNA_Replication_Factors	0.083387692	0.05813

Supplementary Table 1.

Table showing the correlation and associated p-value of HORMAD1 with DNA repair pathways shown in Fig. 5f. A Pearson correlation and significance was derived between HORMAD1 RNA expression and the signature z-score of each DNA repair pathway indicated.

Gene1	Gene2	Log Odds Ratio	p-Value	Adjusted p-	Tendency
HORMAD1	DDR2	>3	<0.001	<0.001	Co-occurrence
HORMAD1	NF1	0.761	0.026	1	Co-occurrence
HORMAD1	ATM	0.68	0.06	1	Co-occurrence
HORMAD1	AKT1	0.775	0.071	1	Co-occurrence
HORMAD1	EML4	0.77	0.086	1	Co-occurrence
HORMAD1	KEAP1	0.548	0.099	1	Co-occurrence
HORMAD1	SMARCA4	0.699	0.107	1	Co-occurrence
HORMAD1	EGFR	-0.556	0.113	1	Mutual exclusivity
HORMAD1	DDR1	0.594	0.131	1	Co-occurrence
HORMAD1	STK11	0.348	0.212	1	Co-occurrence
HORMAD1	RBM10	0.452	0.22	1	Co-occurrence
HORMAD1	ARID1B	0.376	0.225	1	Co-occurrence
HORMAD1	BRAF	0.337	0.254	1	Co-occurrence
HORMAD1	KRAS	0.268	0.256	1	Co-occurrence
HORMAD1	ARID1A	0.299	0.284	1	Co-occurrence
HORMAD1	CCND1	0.643	0.295	1	Co-occurrence
HORMAD1	ARID2	0.296	0.298	1	Co-occurrence
HORMAD1	PIK3CA	-0.309	0.307	1	Mutual exclusivity
HORMAD1	ALK	0.364	0.316	1	Co-occurrence
HORMAD1	CDKN2A	0.22	0.331	1	Co-occurrence
HORMAD1	CDK4	-0.254	0.374	1	Mutual exclusivity
HORMAD1	SETD2	0.207	0.383	1	Co-occurrence
HORMAD1	U2AF1	0.234	0.404	1	Co-occurrence
HORMAD1	CCNE1	0.229	0.428	1	Co-occurrence
HORMAD1	MET	-0.166	0.428	1	Mutual exclusivity
HORMAD1	TP53	-0.105	0.436	1	Mutual exclusivity
HORMAD1	FGFR1	0.174	0.449	1	Co-occurrence
HORMAD1	MYC	0.159	0.476	1	Co-occurrence
HORMAD1	RET	-0.251	0.491	1	Mutual exclusivity
HORMAD1	ERBB2	0.086	0.506	1	Co-occurrence
HORMAD1	NFE2L2	-0.318	0.51	1	Mutual exclusivity
HORMAD1	NRAS	0.092	0.523	1	Co-occurrence
HORMAD1	ROS1	0.092	0.523	1	Co-occurrence
HORMAD1	MDM2	-0.057	0.544	1	Mutual exclusivity
HORMAD1	FBXW7	0.008	0.606	1	Co-occurrence

Supplementary Table 2

Table showing the mutual co-occurrence or exclusivity of significantly mutated genes in the LUAD dataset with HORMAD1 alterations. A log odds ratio and associated p-value was calculated between alterations (mutations and copy number variations) in HORMAD1 and other significantly mutated genes (as defined by TCGA).
